# Supplementary material for: Young adults’ perceptions of living with atopic dermatitis in relation to the concept of self-management: a qualitative study
Source: BMJ Open. 2021 Jun 23;11(6):e044777. doi: 10.1136/bmjopen-2020-044777 (PMC8231057; doi:10.1136/bmjopen-2020-044777)
Supplement: Author's manuscript [file bmjopen-2020-044777.draft_revisions.pdf]

BMJ Open

BMJ Open is committed to open peer review. As part of this commitment we make the peer review history of every article we publish publicly available.

When an article is published we post the peer reviewers' comments and the authors' responses online. We also post the versions of the paper that were used during peer review. These are the versions that the peer review comments apply to.

The versions of the paper that follow are the versions that were submitted during the peer review process. They are not the versions of record or the final published versions. They should not be cited or distributed as the published version of this manuscript.

BMJ Open is an open access journal and the full, final, typeset and author-corrected version of record of the manuscript is available on our site with no access controls, subscription charges or pay-per-view fees (http://bmjopen.bmj.com).

If you have any questions on BMJ Open's open peer review process please email
info.bmjopen@bmj.com

BMJ Open

Young adults' perceptions of living with atopic dermatitis in relation to transition - a qualitative study

Journal:	*BMJ Open*
Manuscript ID	bmjopen-2020-044777
Article Type:	Original research
Date Submitted by the Author:	12-Sep-2020
Complete List of Authors:	Lundin, Susanne; Karolinska Institute,<br>Jonsson, M.; Karolinska Institute Department of Clinical Science and Education Södersjukhuset<br>Fredrik, Carl; Karolinska Institute Department of Medicine Solna<br>Johansson, Emma; Karolinska Institute Department of Medicine Solna<br>Bergstrom, Anna; Karolinska Institute Institute of Environmental Medicine<br>Kull, Inger; Karolinska Institute Department of Clinical Science and Education Södersjukhuset, Karolinska Institutet
Keywords:	Eczema < DERMATOLOGY, QUALITATIVE RESEARCH, Adult dermatology < DERMATOLOGY

SCHOLARONE™
Manuscripts

I, the Submitting Author has the right to grant and does grant on behalf of all authors of the Work (as defined in the below author licence), an exclusive licence and/or a non-exclusive licence for contributions from authors who are: i) UK Crown employees; ii) where BMJ has agreed a CC-BY licence shall apply, and/or iii) in accordance with the terms applicable for US Federal Government officers or employees acting as part of their official duties; on a worldwide, perpetual, irrevocable, royalty-free basis to BMJ Publishing Group Ltd ("BMJ") its licensees and where the relevant Journal is co-owned by BMJ to the co-owners of the Journal, to publish the Work in this journal and any other BMJ products and to exploit all rights, as set out in our licence.

The Submitting Author accepts and understands that any supply made under these terms is made by BMJ to the Submitting Author unless you are acting as an employee on behalf of your employer or a postgraduate student of an affiliated institution which is paying any applicable article publishing charge ("APC") for Open Access articles. Where the Submitting Author wishes to make the Work available on an Open Access basis (and intends to pay the relevant APC), the terms of reuse of such Open Access shall be governed by a Creative Commons licence – details of these licences and which Creative Commons licence will apply to this Work are set out in our licence referred to above.

Other than as permitted in any relevant BMJ Author's Self Archiving Policies, I confirm this Work has not been accepted for publication elsewhere, is not being considered for publication elsewhere and does not duplicate material already published. I confirm all authors consent to publication of this Work and authorise the granting of this licence.

For peer review only

Young adults' perceptions of living with atopic dermatitis in relation to transition - a qualitative study

Short title: Living with eczema in relation to transition

Authors: *Susanne Lundin, PhD-student, RN* [a, b], *Marina Jonsson, PhD* [a, c, f], *Carl-Fredrik Wahlgren, Professor, PhD* [d], *Emma Johansson, PhD* [d, e], *Anna Bergström, PhD* [c, f] *& Inger Kull, Professor, PhD* [a, b]

Affiliations:

[a] Department of Clinical Science and Education, Södersjukhuset, Karolinska Institutet, 118 67 Stockholm, Sweden.

[b] Sachs' Children and Youth Hospital, Södersjukhuset, 118 83 Stockholm, Sweden.

[c] Centre of Occupational and Environmental Medicine, Stockholm County Council, 113 65 Stockholm, Sweden.

[d] Department of Medicine, Solna, Karolinska Institutet, 171 77 Solna, Sweden.

[e] Dermatological and Venereal Clinic, Södersjukhuset, 118 81 Stockholm, Sweden.

[f] Institute of Environmental Medicine, Karolinska Institutet, 171 77 Solna, Sweden.

Corresponding Author: Susanne.lundin@ki.se

Susanne Lundin, Department of Clinical Science and Education, Södersjukhuset, Karolinska Institutet, Jägargatan 20, SE-118 67 Stockholm, Sweden, Tel: +46 707 730 124.

Authors Contributions: SL, MJ and IK contributed to conception and design of the study. The interviews were managed by SL. The analysis with Systematic Text condensation was conducted by SL, MJ and IK in collaboration. Drafting of the manuscript were conducted by SL with support of IK; C-F. W; EJ and AB. All authors participated in critical revision of the manuscript, provided important intellectual input, and approved the final version.

Acknowledgement: We thank all the participants in the BAMSE cohort, and all the staff involved in the study through the years.

For peer review only - http://bmjopen.bmj.com/site/about/guidelines.xhtml

For peer review only

Funding: Funding for this study was provided by the Swedish Asthma and Allergy Association and The Swedish Association for Allergology (SFFA). The funder has not been involved in the design and conduct of the study; collection, management, analysis, and interpretation of the data; preparation, review, or approval of the manuscript; and decision to submit the manuscript for publication.

Conflict of interest: E. Johansson reports personal lecture fees from Sanofi-Genzyme, LEO Pharma, Novartis, ACO, and Galenica. E. Johansson has also been part of the advisory board at Sanofi-Genzyme. C-F. Wahlgren reports grants from Swedish Skin Foundation, during the conduct of the study; grants from Swedish Skin Foundation, outside the submitted work. The rest of the authors declare no relevant conflict of interest

Patient consent: Not required. All participants were orally informed that the results of the study would be published in a scientific journal. Details has been removed from case description to ensure anonymity.

Data sharing statement: No data are available. Supplement material has been deposited anonymised at the BAMSE-secretariat, Institute of Environmental Medicine at Karolinska Institutet, Stockholm, Sweden.

Word Count: 3609/4000

For peer review only - http://bmjopen.bmj.com/site/about/guidelines.xhtml

Abstract

Objectives:

Learning to take control of one's health is an important part of the transition from adolescence to adulthood. This study aimed to explore young adults' perceptions of living with atopic dermatitis (AD) in relation to the transition process.

Design:

A qualitative study with an inductive approach was performed through semi-structured interviews (n=15). The interviews were recorded, transcribed verbatim and analysed with systematic text condensation.

Participants:

Young adults (22-24y) with persistent atopic dermatitis in a longitudinal population-based birth cohort. To capture experience of living with persistent AD (preschool/school-age onset) of different severity (mild to very severe), a strategic selection was performed. In total 15 young adults were included. Persistent AD was defined as preschool/school-age onset with dry skin in combination with itchy rash of typical localisation. Severity was self-assessed using the Patient Oriented Eczema Measure.

Results:

Despite having experience of AD since childhood, the respondents expressed uncertainty about treatment and how it affected their bodies. Their uncertainties and feelings affected how they used TGCs. The respondents emphasised that they perceived availability of healthcare and knowledge about treatment of AD among healthcare providers to be limited. The participants did not state any experiences of transfer from paediatric to adult healthcare, which affect young adults' possibilities to take full control of their AD care.

For peer review only

Conclusions:

Young adults with preschool/school-age onset of AD are unsure how to treat and manage the disease. One explanation may be insufficient transition process.

Key words: Atopic dermatitis, Interviews, Self-management, Transition, Young adults

Word count, 236/300

For peer review only

For peer review only - http://bmjopen.bmj.com/site/about/guidelines.xhtml

Article summary

Strength and limitation

- Data from a longitudinal population-based birth cohort
- The respondent had well-defined AD based on both prospectively recorded questionnaire data and clinical examinations.
- The AD severity ranged from mild to very severe, measured by POEM, a validated questionnaire.
- Equal representation of young men and women.
- The results may not be transferred to young adults with more severe atopic dermatitis demanding in specialist care.

For peer review only

For peer review only - http://bmjopen.bmj.com/site/about/guidelines.xhtml

Introduction

To optimise self-management in young people with chronic disease, there is a need for a transition process to increase knowledge and to achieve self-care skills and independence [1, 2]. To aid young adults to be autonomous and minimizing dependency from parents and healthcare regarding self-management of eczema, it is important that the transition process starts at an early age (11–13 years) in collaboration with the family and all stakeholders [3]. Atopic dermatitis (AD) [4] is a chronical disease that cannot be cured as yet and requires daily treatment [5]. The symptoms range from mild to very severe [6]. Most individuals with AD have a mild disease [7, 8], but there seems to be a discordance between patients' and physicians' assessments of severity [9, 10]. Moreover, it is well known that eczema affects health-related quality of life (HRQoL) in both children and adults [7, 11].

The goals of treatment are to increase disease control and to extend asymptomatic periods as much as possible [7, 12]. The mainstay of treatment is use of emollients to decrease dryness and extend time to relapse of AD [6] and the first-line treatment of inflammation consists of topical glucocorticoids (TGCs) [12]. The chronic recurrent nature of the disease [7] and the often time-consuming nature of treatment can be challenge for individuals with AD [13]. Since parents have the main responsibility for the treatment of AD in their children, it is of importance that they have been trained to and understand the best management of the disease [6], a shift in responsibility takes place when the children reach adolescence, which requires that the adolescents gradually develop knowledge of their treatment. However, results from a Swedish population-based birth cohort shows that many adolescents with AD are untreated [14].

Special transition programmes have been developed for some chronic diseases, but not – to our knowledge – for young persons with AD. According to European Academy of Allergy and Clinical Immunology recently published guidelines on effective transition [3], learning to take control of treatment is an important part of the transition from adolescence to adulthood.

For peer review only - http://bmjopen.bmj.com/site/about/guidelines.xhtml

Overall experiences of eczema and transition are scarcely studied. The aim of this qualitative study was therefore to explore young adults' perceptions of living with AD in relation to the transition process.

Methods

Design

A qualitative semi-structured interview study with an inductive approach was used to capture young adults' experiences of AD and transition. We followed the Consolidated Criteria for Reporting Qualitative Research guideline for conducting and reporting qualitative research [15]

Setting

The participants were recruited from the longitudinal population-based cohort BAMSE, to which they had been recruited as new-borns, from four predefined areas in Stockholm [16]. Their parents responded to questionnaires about family history of allergic/atopic disease and various lifestyle factors. Follow-up questionnaires about symptoms of allergic/atopic diseases were answered by the parents at participant age 1, 2, 4, and 8 years, by both the parents and the adolescents at participant age 12 and 16 years, and by the young adults at age 24 years. A clinical examination was performed at the 24-years follow-up. To capture young adults with experience of persistent AD (preschool/school-age onset), with various symptoms (mild to very severe), a strategic selection was performed. At time for inclusion, 66 young adults with AD were eligible. After the clinical examination, every third of these participants was invited to the interview study from a random list by a study nurse over the telephone.

For peer review only - http://bmjopen.bmj.com/site/about/guidelines.xhtml

Inclusion criteria

Young adults with persistent AD, defined as dry skin in combination with itchy rash of typical localisation (neck/throat, arm/leg flexures and/or wrists/ankles) in the 12 months preceding the 16- and the 24-year follow-up. At the 24-year follow-up, the severity of the disease was self-assessed as mild to very severe AD using the Patient Oriented Eczema Measure [17], which enabled for us to capture a range of experiences. The young adults who fulfilled both the questionnaire definition of AD (as above) and the clinical definition of AD, as assessed by a trained nurse: visible AD on the neck/throat and/or flexure surfaces of the arms/legs and/or wrists/ankles and/or reported symptoms of itchy skin in the preceding week were included.

Data collection

All interviews were conducted at the research clinic, except two, which were conducted by telephone. The interviews were performed by an experienced nurse working at the paediatric dermatology unit. The semi-structured interview guide consisted of five themes related to living with AD: daily life, symptoms, treatment, care, and consequences of the disease (Table 1). Probing questions were used to get a deeper understanding or explanation of some statements. To test the interview guide, one pilot interview was conducted. The interviews lasted on average 31 (range 23–43) minutes. Data collection continued until data saturation was judged to have been achieved. In total, 15 young adults (table 2) were included, which gave us rich data covering the purpose of the study. All interviews were recorded and transcribed verbatim.

For peer review only - http://bmjopen.bmj.com/site/about/guidelines.xhtml

Data analysis

The text was analysed by three researchers in collaboration using systematic text condensation, a thematic cross-case strategy in four steps [18]. First, all interviews were read through, and preliminary themes were identified based on the contents of the text. In the second step, meanings units were identified based on the contents of the preliminary themes and code groups were developed. In the third step, the material from each code group was decontextualized, sorted into subgroups and the contents of the text were condensed. Lastly, in the fourth step, data were reconceptualised, and categories made based on the essence of the young adults' experiences of eczema (Figure 1). The analysis was inductive. We used an editing analysis style where categories were developed from empirical data.

Ethics

The study was approved by the regional ethical review board at Karolinska Institutet, Stockholm (approval number 2016/1380-31-2, 2017/395-32). All participants gave informed consented, 13 with written consent and two by telephone.

Patient and public involvement

The primary intention of this study was to capture young adults' perceptions of living with atopic dermatitis in relation to the transition process. However, the young adults were not involved in the design of the study.

For peer review only

For peer review only - http://bmjopen.bmj.com/site/about/guidelines.xhtml

Results

The respondents' perceptions of living with AD in the transition process resulted in four categories and seven subgroups (Figure 2). Despite having had AD since childhood, the respondents expressed uncertainty about treatment and how it could affect their bodies. Moreover, both symptoms and treatment were described as painful. Their uncertainties and feelings affected how they used TGCs. Though the disease made itself known all the time, most of the participants stated that they had gotten used to living with eczema. The respondents emphasised that they experienced that both the availability of healthcare and the knowledge about treatment of AD among healthcare providers were limited. This indicates insufficiencies in transition, which affect young adults' possibilities to take full control of their AD care.

Individual understanding and knowledge

Although most of the respondents had had AD from an early age, their knowledge about its cause and treatment was limited. They had created their own treatment routines, based on their experiences and/or what they have learned from their parents. The young adults stated that they experienced a lack of support and directives on treatment.

Ignorance

The young adults expressed ignorance about the cause of their AD, how they should treat it, what they should use to treat it, where on the body, and for how long. They had not received much information about the cause or variability of the disease, or about different treatments.

"The doctors have been more like it will go away when you reach a certain age or something, it's just now, it will disappear, so ... I'm just waiting for that age to come" (9)

For peer review only

They felt uncertain about the treatment, if they were doing the right thing, and if the topical treatment should include or consist of cortisone.

"They say cortisone is what helps, I don't know, there's no cortisone in Mometasone, as far as I know" (3)

There was a general belief among respondents that cortisone attenuated or diminished symptoms only for the moment, which reinforced the belief that they could have "become immune" to cortisone creams.

Most of the young adults said they treated themselves regularly, but it was clear they were primarily referring to moisturising creams. The respondents mentioned different treatment recommendations they had been given over the years, such as not to use cortisone around the eyes and not to apply cream if it was cold outside. They had also been advised to use cortisone sparingly, at most twice a week, without regard to the severity of the AD.

The respondents said that it would be helpful with more information and requested a guide with treatment principles, depending on the severity of the AD, which would be important in the transition process, to achieve independence regarding management of the disease.

"I have understood by, like, reading and talking with different people that you should have different, like, cycles for it, but I haven't really understood how to do it, because I have not got that input from a doctor, how I should do it" (14)

Impact on my body

Both symptoms and treatment of the AD had a painful impact on the body. Moreover, the need to use different creams to treat the AD was something that felt unnatural and there were thoughts on various treatments adversely affecting the body.

For peer review only - http://bmjopen.bmj.com/site/about/guidelines.xhtml

It hurts

Many of the respondents stated that one of the most difficult things about AD was that it hurt. They described the pain as a burning and stinging feeling under the skin. That pain was annoying and could prevent them from doing things that they liked to do. The pain was sometimes more trying than the itching.

"Periodically, it hurt too much to put on socks and shoes…" (1)

They stated that it was painful to apply cream when the AD was severe; they self-treated to get rid of the pain, but that hurt even more.

"It stings, of course, every time I apply the cream" (15)

Unnatural

Some of the respondents felt that it was unnatural to cover their skin with something that could affect the body negatively, especially in the case of cortisone.

"That you absorb, like, products that the body is not really made to deal with" (2)

They had heard that the skin could become damaged and thinner from using cortisone, which made them anxious; they therefore used cortisone creams as little as possible. They felt that it was strange to use a treatment that could simultaneously damage the skin even if it decreased the AD.

"I try not to use too much, plain and simple, and hope it is…that I'm like doing what you're supposed to" (7)

Availability and knowledge in healthcare

There was a perception among the young adults that it was difficult to get help from healthcare, both making an appointment and getting the "right" treatment. The respondents

For peer review only

did not state any experiences of transfer from paediatric to adult healthcare. Moreover, they perceived that healthcare providers did not recognise the symptoms, which resulted in insufficient and delayed treatment.

Difficult to get help

Even though most of the respondents had had AD all their life, they stated that they had limited contact with healthcare. It seemed to be difficult to get in touch with healthcare and get appropriate help, even when the AD was severe. Sometimes, it took several months to get an appointment and they needed to spend a lot of time trying to get in touch with the care provider. Many of the respondents could not remember the last time they had had an appointment.

"It was probably in August that I contacted my dermatology department, because it [i.e., eczema] was so very bad…and then I got an appointment for the middle of November" (14)

One respondent had experienced that the symptoms of the AD had not been taken seriously; the physician downplayed the respondent's symptoms and compared them with the more widespread symptoms of others. The participants perceived that physicians in general had limited knowledge about treatment of AD and the only advice they had received was to apply topical creams, without further instructions. Information about how they should treat the eczema also differed from physician to physician.

"It seems that the GP doesn't know all that much about eczema…and just prescribes ointments that do not help" (8)

"Some of them say 'apply it when you need to,' others say, 'apply it like several times a day,' it's like people are guessing a little, they don't have any idea, really" (10)

For peer review only - http://bmjopen.bmj.com/site/about/guidelines.xhtml

Get used to it

The respondents stated that they had become accustomed to living with AD and had accepted their disease. Even so, they had many thoughts about how they should avoid worsening of the AD, which required a lot of discipline in the form of regular treatment, avoiding scratching, and creating strategies in daily life.

Thoughts

Most of the respondents thought of and were aware of their AD constantly: when it was itching, when it was visible, when they had to apply cream, and so on. They also had strategies to avoid worsening of the AD. The respondents perceived it as trying to experience itchiness all the time and they were aware of the importance of not scratching, even though it was difficult. Trying not to scratch required constant vigilance. Scratching was the only thing they wanted to do, but they knew it would immediately result in them being "punished", through worsening of the AD.

"That's the main thing in how I handle it, that just sitting there thinking that okay, don't scratch, don't scratch, don't scratch, don't scratch, don't scratch, it has become like a habit to try not to do it" (15)

The major problem was that the AD never disappeared. They had to make choices in their daily life based on their skin condition. Most of the respondents never felt free of symptoms, which they found trying.

"It's probably all the time I'd say, on different parts, it's like it moves around a bit, where it's, like, worst" (10)

For peer review only - http://bmjopen.bmj.com/site/about/guidelines.xhtml

Discipline

The treatment of AD was demanding, according to the respondents. Treatment was a bit awkward and nothing they enjoyed. They had to have a lot of discipline; even then, it took a long time to get rid of the AD.

"But the eczema keeps, like, coming back anyway, even though I have this routine" (9)

The respondents stated that the creams only helped for the moment and some believed that they had "become immune" to them. They always had to carry creams with them, to school, work, or when they travelled, and if they forgot they were "punished" at once, through worsening of the AD.

"Always having creams with you and applying them is not all that much fun either" (3)

Acceptance

The respondents emphasised that they had gotten used to living with AD and had accepted their situation, even though it was hard. They did not allow AD to keep them from doing things.

"It's, like, something you have, you have to learn to live with it, maybe" (7)

While they had accepted their disease, they tried to avoid revealing their AD, in so far as possible, when it was in visible places on their bodies.

"I do not like it to be visible, so usually when I have these red, I usually use long sleeves, to like cover up" (13)

For peer review only

For peer review only - http://bmjopen.bmj.com/site/about/guidelines.xhtml

Discussion

This qualitative interview study of young adults with AD from the general population demonstrated several shortcomings in the transition process. Even though the young adults had experience of persistent AD, they were unsure about how to treat the AD. The respondents expressed ignorance about the differences between emollients and TGCs and regarding the potency of the latter. Their uncertainty may be related to their perception of difficulties in getting help from healthcare and of limited knowledge about treatment of AD among healthcare providers. While acceptance of the disease was high, the respondents never felt completely symptom-free. Altogether, this indicates an insufficient transition process, regarding treatment and management of the disease.

Knowledge about treatment of eczema

The young adults' uncertainty regarding how to treat and manage the AD was in accordance with other studies. The uncertainty seems to be found also in self-treating adults, even if their AD is more severe; this can delay the start of treatment in flare-ups [19]. In contrast, adolescents in a qualitative study [20] expressed satisfaction with the effect of their treatments, even though half of them used TGCs every day because of low efficiency. Moreover, they had limited knowledge and incorrect beliefs about how the treatment worked. The same beliefs were seen in the present study, but the participants in our study expressed less satisfaction and more often believed that treatment, even with emollients, could harm their body.

The transition process

The limited contact with and difficulties in getting help from healthcare can to some extent be due to limited knowledge regarding whom to contact. Healthcare in Sweden is divided into primary care, specialist care, and highly specialised care [21]. Primary care is responsible for treatment and care of the most common skin diseases, such as AD. Individuals with AD can

For peer review only - http://bmjopen.bmj.com/site/about/guidelines.xhtml

buy up to 30 g of mild TGCs over the counter from a pharmacy; for larger amounts or more potent TGCs, a prescription is needed. The respondents' opinions that physicians had limited knowledge about treatment is in accordance with the results from a qualitative study including GPs. The GPs stated that they used a trial-and-error approach when prescribing emollients, due to the large assortment of different creams, and that they were reluctant to prescribe strong TGCs to children [22]. The prescribed amounts of emollients in primary care have reportedly been unexpectedly low when compared with guidelines, which was also seen for prescription of TGCs in specialist care [23, 24].

Concerns about side effects of TGCs is common. Studies including both children and adults with moderate to severe AD have found that approximately 50% of guardians/patients are reluctant to use TGCs [25, 26]. In addition, misleading information from relatives/friends, the internet, or pharmacies can lead to poor adherence [27, 28].

Moreover, it has been shown that concerns and uncertainties about side effects exist even among healthcare providers [29]. Both GPs and pharmacists inform patients about the risks of TGCs and that they should be applied sparingly and not over long periods of time [30]. This can affect the strength and amount of TGCs prescribed, and strengthen the feeling among individuals with AD that cortisone is something to be careful with [31, 32].

While the young adults in the present study had gotten used to living with AD, they stated that they needed more support. Furthermore, there seemed to be a need for repeated information about the cause, course, and treatment of the disease. Several studies have stated the importance of shared decision-making, education, and a written action plan [33-36], which could be useful tools to achieve independence. This is important in the process of developing autonomy in the transition process, and has been confirmed in a study among youths aged 14–24 years [37], which showed that 41% wanted to be more involved in their care. For adolescents with asthma, it is known that support during the transition process results in better adherence

For peer review only - http://bmjopen.bmj.com/site/about/guidelines.xhtml

to treatment, but the healthcare transition in AD is less studied [38]. Guidelines on asthma emphasise the importance of reviewing each patient's inhalation technique at every healthcare visit, to decrease the risk of exacerbations [39]. It would be equally important to review how patients with AD perform self-treatment, to achieve as long symptom-free periods as possible.

Strengths and limitations

One strength of the present study was that all respondents had well-defined AD based on both prospectively recorded questionnaire data and clinical examinations. Moreover, their AD severity ranged from mild to very severe. Additional strengths are the credibility and transferability based on the wide range of data from the respondents, which we have exemplified using the respondents' own words.

The respondents were from the general population, which is a strength since most patients with AD are treated in primary care. However, the results may not be transferred to patients with more severe AD, who may have more contact with specialist care. Still, some of the respondents in the present study had moderate to very severe AD.

In qualitative studies, the number of participants is generally low. However, this can be compensated by a strategic selection, to get a deeper understanding of the respondents' experiences. We recruited participants with long experience of mild to very severe AD, who wanted to share their experiences. The data collection gave us varied and rich data on perceptions and experiences of living with AD. An interviewer's preconception about subjects can be both positive and negative. Deep experience can be helpful in the interview situation to promote understanding and when asking probing questions, but can also be negative, leading to assumed understanding of what informants are saying and an assumption that the interviewer and respondent are thinking about the same thing. Preconceptions can also be

For peer review only - http://bmjopen.bmj.com/site/about/guidelines.xhtml

negative during the analysis phase. To minimise these effects, the analysis was carried out in collaboration with two co-authors with little or no experience in the care of patients with AD.

Conclusions

This qualitative interview study of young adults with AD from the general population demonstrated several shortcomings in the transition process. Despite long experience of AD, the young adults expressed ignorance and uncertainty about treatment. Their uncertainty may be related to their perception of difficulties in getting help from healthcare and of limited knowledge about treatment of AD among healthcare providers.

For peer review only

For peer review only - http://bmjopen.bmj.com/site/about/guidelines.xhtml

References

1. Gabriel P, McManus M, Rogers K, White P. Outcome Evidence for Structured Pediatric to Adult Health Care Transition Interventions: A Systematic Review. *The Journal of pediatrics*. Sep 2017;188:263-269.e15. doi:10.1016/j.jpeds.2017.05.066
2. Schmidt A, Ilango SM, McManus MA, Rogers KK, White PH. Outcomes of Pediatric to Adult Health Care Transition Interventions: An Updated Systematic Review. *Journal of pediatric nursing*. Mar-Apr 2020;51:92-107. doi:10.1016/j.pedn.2020.01.002
3. Roberts G, Vazquez-Ortiz M, Knibb R, et al. EAACI Guideline on the effective transition of adolescents and young adults with allergy and asthma. *Allergy*. Jun 19 2020;doi:10.1111/all.14459
4. Kantor R, Thyssen JP, Paller AS, Silverberg JI. Atopic dermatitis, atopic eczema, or eczema? A systematic review, meta-analysis, and recommendation for uniform use of 'atopic dermatitis'. *Allergy*. Oct 2016;71(10):1480-5. doi:10.1111/all.12982
5. Bieber T. Atopic dermatitis. *Annals of dermatology*. May 2010;22(2):125-37. doi:10.5021/ad.2010.22.2.125
6. Wollenberg A, Barbarot S, Bieber T, et al. Consensus-based European guidelines for treatment of atopic eczema (atopic dermatitis) in adults and children: part I. *Journal of the European Academy of Dermatology and Venereology : JEADV*. May 2018;32(5):657-682. doi:10.1111/jdv.14891
7. Weidinger S, Novak N. Atopic dermatitis. *Lancet*. Mar 12 2016;387(10023):1109-22. doi:10.1016/S0140-6736(15)00149-X
8. Ballardini N, Kull I, Soderhall C, Lilja G, Wickman M, Wahlgren CF. Eczema severity in preadolescent children and its relation to sex, filaggrin mutations, asthma, rhinitis, aggravating factors and topical treatment: a report from the BAMSE birth cohort. *Br J Dermatol*. Mar 2013;168(3):588-94. doi:10.1111/bjd.12196
9. Wei W, Anderson P, Gadkari A, et al. Discordance Between Physician- and Patient-Reported Disease Severity in Adults with Atopic Dermatitis: A US Cross-Sectional Survey. *American journal of clinical dermatology*. Dec 2017;18(6):825-835. doi:10.1007/s40257-017-0284-y
10. Holm EA, Wulf HC, Stegmann H, Jemec GB. Life quality assessment among patients with atopic eczema. *Br J Dermatol*. Apr 2006;154(4):719-25. doi:10.1111/j.1365-2133.2005.07050.x
11. Grant L, Seiding Larsen L, Trennery C, et al. Conceptual Model to Illustrate the Symptom Experience and Humanistic Burden Associated With Atopic Dermatitis in Adults and Adolescents. *Dermatitis : contact, atopic, occupational, drug*. Jul/Aug 2019;30(4):247-254. doi:10.1097/der.0000000000000486
12. Weidinger S, Beck LA, Bieber T, Kabashima K, Irvine AD. Atopic dermatitis. *Nature reviews Disease primers*. Jun 21 2018;4(1):1. doi:10.1038/s41572-018-0001-z
13. Santer M, Burgess H, Yardley L, et al. Managing childhood eczema: qualitative study exploring carers' experiences of barriers and facilitators to treatment adherence. *Journal of advanced nursing*. Nov 2013;69(11):2493-501. doi:10.1111/jan.12133
14. Lundin S, Wahlgren CF, Bergstrom A, et al. Use of emollients and topical glucocorticoids among adolescents with eczema - datafrom the population-based birth cohort BAMSE. *Br J Dermatol*. Feb 25 2018;doi:10.1111/bjd.16484
15. Tong A, Sainsbury P, Craig J. Consolidated criteria for reporting qualitative research (COREQ): a 32-item checklist for interviews and focus groups. *International journal for quality in health care : journal of the International Society for Quality in Health Care*. Dec 2007;19(6):349-57. doi:10.1093/intqhc/mzm042
16. Wickman M, Kull I, Pershagen G, Nordvall SL. The BAMSE project: presentation of a prospective longitudinal birth cohort study. *Pediatr Allergy Immunol*. 2002;13 Suppl 15:11-3.
17. Charman CR, Venn AJ, Williams HC. The patient-oriented eczema measure: development and initial validation of a new tool for measuring atopic eczema severity from the patients' perspective. *Arch Dermatol*. Dec 2004;140(12):1513-9. doi:10.1001/archderm.140.12.1513
18. Malterud K. Systematic text condensation: a strategy for qualitative analysis. *Scandinavian journal of public health*. Dec 2012;40(8):795-805. doi:10.1177/1403494812465030

For peer review only - http://bmjopen.bmj.com/site/about/guidelines.xhtml

19. Ortiz de Frutos FJ, Torrelo A, de Lucas R, et al. Patient perspectives on triggers, adherence to medical recommendations, and disease control in atopic dermatitis: the DATOP study. *Actas dermo-sifiliograficas*. Jun 2014;105(5):487-96. doi:10.1016/j.ad.2014.01.004
20. Kosse RC, Bouvy ML, Daanen M, de Vries TW, Koster ES. Adolescents' Perspectives on Atopic Dermatitis Treatment-Experiences, Preferences, and Beliefs. *JAMA Dermatol*. Jul 1 2018;154(7):824-827. doi:10.1001/jamadermatol.2018.1096
21. Council SR. The Swedish healthcare system. Updated 2020-02-25. Accessed 2020-07-01,
22. Le Roux E, Powell K, Banks JP, Ridd MJ. GPs' experiences of diagnosing and managing childhood eczema: a qualitative study in primary care. *The British journal of general practice : the journal of the Royal College of General Practitioners*. Feb 2018;68(667):e73-e80. doi:10.3399/bjgp18X694529
23. Choi JY, Dawe R, Ibbotson S, Fleming C, Doney A, Foerster J. Topical treatments in atopic dermatitis: unexpectedly low use of emollients; use of topical corticosteroid is higher in juvenile patients, higher in male vs females, and shows independent associations with asthma and depression. *Br J Dermatol*. Jun 30 2019;doi:10.1111/bjd.18265
24. Schmitt J, Schmitt NM, Kirch W, Meurer M. Outpatient care and medical treatment of children and adults with atopic eczema. *Journal der Deutschen Dermatologischen Gesellschaft = Journal of the German Society of Dermatology : JDDG*. Apr 2009;7(4):345-51. doi:10.1111/j.1610-0387.2008.06967.x
25. Zuberbier T, Orlow SJ, Paller AS, et al. Patient perspectives on the management of atopic dermatitis. *J Allergy Clin Immunol*. Jul 2006;118(1):226-32. doi:10.1016/j.jaci.2006.02.031
26. Jung HJ, Bae JY, Kim JE, et al. Survey of disease awareness, treatment behavior and treatment satisfaction in patients with atopic dermatitis in Korea: A multicenter study. *The Journal of dermatology*. Oct 2018;45(10):1172-1180. doi:10.1111/1346-8138.14540
27. Smith SD, Farrugia LL, Harris V, et al. Evaluation of the influence of family and friends, and the Internet on patient perceptions of long-term topical corticosteroid use. *The Journal of dermatological treatment*. Nov 2017;28(7):642-646. doi:10.1080/09546634.2017.1306017
28. Smith SD, Hong E, Fearns S, Blaszczynski A, Fischer G. Corticosteroid phobia and other confounders in the treatment of childhood atopic dermatitis explored using parent focus groups. *The Australasian journal of dermatology*. Aug 2010;51(3):168-74. doi:10.1111/j.1440-0960.2010.00636.x
29. Bos B, Antonescu I, Osinga H, Veenje S, de Jong K, de Vries TW. Corticosteroid phobia (corticophobia) in parents of young children with atopic dermatitis and their health care providers. *Pediatric dermatology*. Jan 2019;36(1):100-104. doi:10.1111/pde.13698
30. Farrugia LL, Lee A, Fischer G, Blaszczynski A, Carter SR, Smith SD. Evaluation of the influence of pharmacists and GPs on patient perceptions of long-term topical corticosteroid use. *The Journal of dermatological treatment*. Mar 2017;28(2):112-118. doi:10.1080/09546634.2016.1213353
31. Arkwright PD, Motala C, Subramanian H, Spergel J, Schneider LC, Wollenberg A. Management of difficult-to-treat atopic dermatitis. *The journal of allergy and clinical immunology In practice*. Mar 2013;1(2):142-51. doi:10.1016/j.jaip.2012.09.002
32. Feldman SR, Vrijens B, Gieler U, Piaserico S, Puig L, van de Kerkhof P. Treatment Adherence Intervention Studies in Dermatology and Guidance on How to Support Adherence. *American journal of clinical dermatology*. Apr 2017;18(2):253-271. doi:10.1007/s40257-017-0253-5
33. Noerreslet M, Jemec GB, Traulsen JM. Involuntary autonomy: patients' perceptions of physicians, conventional medicines and risks in the management of atopic dermatitis. *Social science & medicine (1982)*. Nov 2009;69(9):1409-15. doi:10.1016/j.socscimed.2009.08.036
34. Stalder JF, Bernier C, Ball A, et al. Therapeutic patient education in atopic dermatitis: worldwide experiences. *Pediatric dermatology*. May-Jun 2013;30(3):329-34. doi:10.1111/pde.12024
35. Muller SM, Tomaschett D, Euler S, Vogt DR, Herzog L, Itin P. Topical Corticosteroid Concerns in Dermatological Outpatients: A Cross-Sectional and Interventional Study. *Dermatology (Basel, Switzerland)*. 2016;232(4):444-52. doi:10.1159/000446068

For peer review only - http://bmjopen.bmj.com/site/about/guidelines.xhtml

36. Shi VY, Nanda S, Lee K, Armstrong AW, Lio PA. Improving patient education with an eczema action plan: a randomized controlled trial. *JAMA Dermatol*. Apr 2013;149(4):481-3. doi:10.1001/jamadermatol.2013.2143
37. Schuiteman S, Chua KP, Plegue MA, Ilyas O, Chang T. Self-Management of Health Care Among Youth: Implications for Policies on Transitions of Care. *The Journal of adolescent health : official publication of the Society for Adolescent Medicine*. Feb 26 2020;doi:10.1016/j.jadohealth.2020.01.009
38. Vazquez-Ortiz M, Angier E, Blumchen K, et al. Understanding the challenges faced by adolescents and young adults with allergic conditions: a systematic review. *Allergy*. Mar 6 2020;doi:10.1111/all.14258
39. GINA gifa. Pocket guide for asthma management and prevention (for adults and children older than 5 years). Updated 2020-02-28.

For peer review only

For peer review only - http://bmjopen.bmj.com/site/about/guidelines.xhtml

Table 1. Interview guide.

Themes related to experiences of eczema, treatment and care	Follow-up questions
Tell me what it is like to live with eczema.	Tell me what it has been like lately.<br>What was it like when you were a teenager?<br>Is there any difference?
Tell me what it is like when you have trouble with your eczema.	What is it like when your eczema is not troubling you?<br>How often do you have trouble with your eczema?<br>What do you think is the cause of your eczema?
Tell me how you usually self-treat.	How do you feel it works?<br>How has treatment been shown or explained to you?<br>What do you think about cortisone?<br>What do you think about moisturizing cream?
Tell me about the experiences you have of healthcare in relation to your eczema.	Tell me about the needs you have from healthcare due to your eczema.<br>Tell me about any information you have received from healthcare regarding your eczema.<br>Could it have been given in any other way?
In what situations do you think about your eczema?	Do you avoid doing anything because of your eczema?<br>Have you avoided anything in the past because of your eczema?

For peer review only

Table 2. Background factors of participants: age, sex, severity according to POEM and onset of eczema.

ID No	Age	Sex	Self-assessed severity at 16 y [1]	Self-assessed severity at 24 y [1]	Onset of eczema up to 4 years [2]	Onset of eczema from 8 years [3]
1	24 y 2 m	M	Mild	Moderate		X
2	23 y 11 m	F	Mild	Moderate	X	
3	23 y 2 m	M	Moderate	Moderate	X	
4	23 y 3 m	M	Mild	Moderate	X	
5	23 y 6 m	M	Mild	Mild		X
6	23 y 5 m	F	Mild	Mild		X
7	24 y 4 m	M	Moderate	Severe/very severe	X	
8	23 y 7 m	M	Moderate	Mild	X	
9	22 y 11 m	F	Mild	Moderate	X	
10	24 y 10 m	F	Mild	Severe/very severe	X	
11	22 y 3 m	F	Severe/very severe	Moderate	X	
12	24 y 3 m	M	Mild	Severe/very severe	X	
13	22 y 3 m	F	Moderate	Moderate	X	
14	24 y 7 m	F	Mild	Severe/very severe		X
15	22 y 11 m	M	Severe/very severe	Severe/very severe	X	

[1] POEM class 1, mild eczema (3–7), class 2, moderate eczema (8–16), class 3, severe/very severe eczema (17–28).

[2] Preschool eczema (i.e. eczema onset at 1,2 or 4, not 8,12 and 16 years of age)

[3] School age eczema (i.e. eczema onset at 8, 12 or 16 not at 1,2 or 4 years of age)

For peer review only

For peer review only - http://bmjopen.bmj.com/site/about/guidelines.xhtml

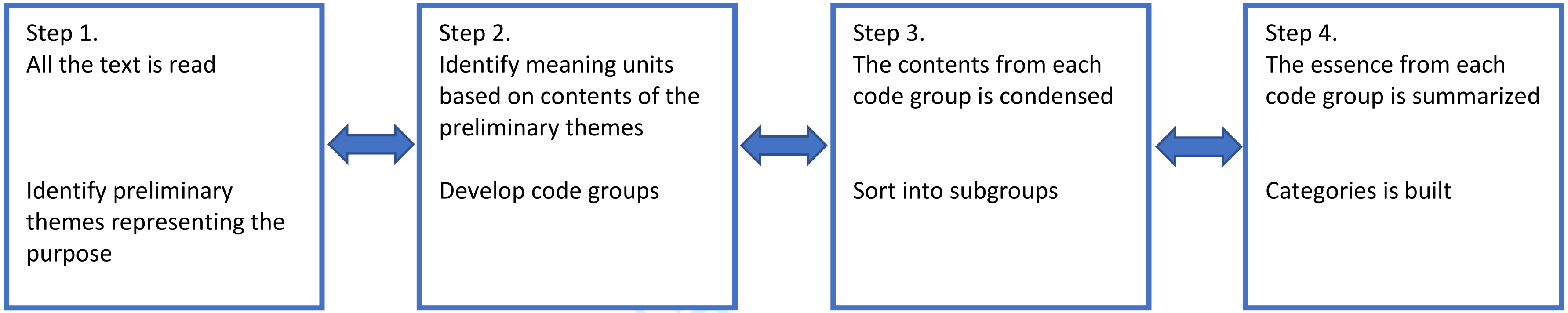

Figure 1. The analysis process of Systematic Text Condensation

For peer review only

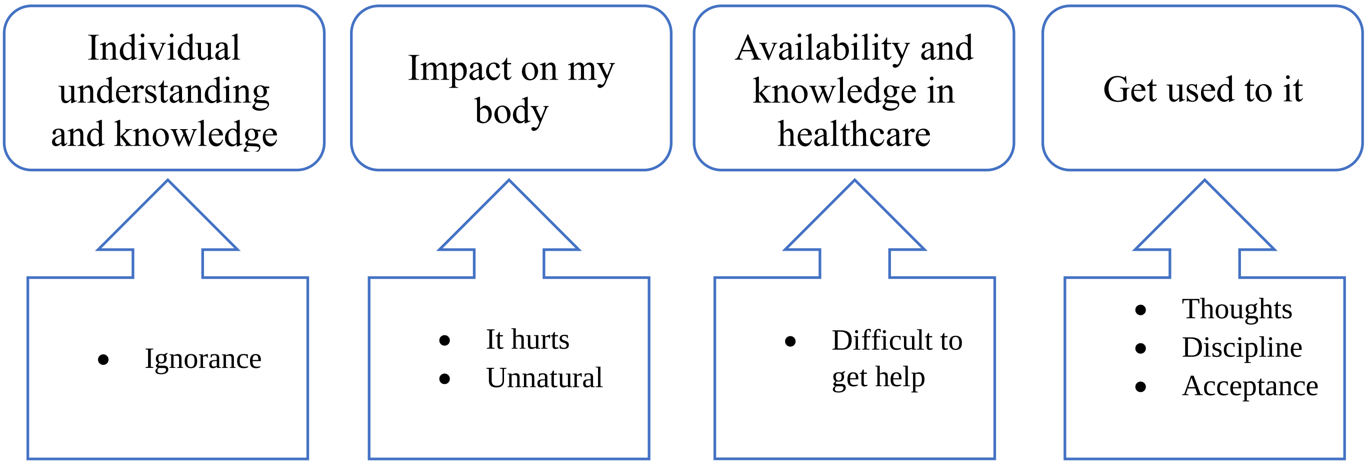

Figure 2. Results in the form of categories and subgroups.

For peer review only

Table 1

Consolidated criteria for reporting qualitative studies (COREQ): 32-item checklist

No	Item	Guide questions/description
**Domain 1: Research team and reflexivity**		
Personal Characteristics		
1.	Interviewer/facilitator	Which author/s conducted the interview or focus group? page 1
2.	Credentials	What were the researcher's credentials? *E.g. PhD, MD* page 1
3.	Occupation	What was their occupation at the time of the study? page 8
4.	Gender	Was the researcher male or female? page
5.	Experience and training	What experience or training did the researcher have? page 8
Relationship with participants		
6.	Relationship established	Was a relationship established prior to study commencement? No
7.	Participant knowledge of the interviewer	What did the participants know about the researcher? e.g. *personal goals, reasons for doing the research* At start of the interviews the author introduce herself as RN + PhD-Student

8.	Interviewer characteristics	What characteristics were reported about the interviewer/facilitator? e.g. *Bias, assumptions, reasons and interests in the research topic* page 8, 18
**Domain 2: study design**		
Theoretical framework		
9.	Methodological orientation and Theory	What methodological orientation was stated to underpin the study? *e.g. grounded theory, discourse analysis, ethnography, phenomenology, content analysis* page 9
Participant selection		
10.	Sampling	How were participants selected? e.g. *purposive, convenience, consecutive, snowball* page 7
11.	Method of approach	How were participants approached? e.g. *face-to-face, telephone, mail, email* page 7
12.	Sample size	How many participants were in the study? page 8
13.	Non-participation	How many people refused to participate or dropped out? Reasons? page 8
Setting		
14.	Setting of data collection	Where was the data collected? e.g. *home, clinic, workplace* page 8
15.	Presence of non-participants	Was anyone else present besides the participants and researchers? No

16.	Description of sample	What are the important characteristics of the sample? *e.g. demographic data, date*	page 8
Data collection			
17.	Interview guide	Were questions, prompts, guides provided by the authors? Was it pilot tested?	page 8
18.	Repeat interviews	Were repeat interviews carried out? If yes, how many?	No
19.	Audio/visual recording	Did the research use audio or visual recording to collect the data?	page 8
20.	Field notes	Were field notes made during and/or after the interview or focus group?	No
21.	Duration	What was the duration of the interviews or focus group?	page 8
22.	Data saturation	Was data saturation discussed?	page 8
23.	Transcripts returned	Were transcripts returned to participants for comment and/or correction?	No
**Domain 3: analysis and findings**z			
Data analysis			
24.	Number of data coders	How many data coders coded the data?	
25.	Description of the coding tree	Did authors provide a description of the coding tree?	page 8 + Figure 1+2

26.	Derivation of themes	Were themes identified in advance or derived from the data?	page 9
27.	Software	What software, if applicable, was used to manage the data?	—
28.	Participant checking	Did participants provide feedback on the findings?	No
Reporting			
29.	Quotations presented	Were participant quotations presented to illustrate the themes / findings? Was each quotation identified? *e.g. participant number*	page 10-15
30.	Data and findings consistent	Was there consistency between the data presented and the findings?	page 10-15
31.	Clarity of major themes	Were major themes clearly presented in the findings?	page 10-15
32.	Clarity of minor themes	Is there a description of diverse cases or discussion of minor themes?	—

Young adults' perceptions of living with atopic dermatitis in relation to the concept of self-management - a qualitative study

Journal:	*BMJ Open*
Manuscript ID	bmjopen-2020-044777.R1
Article Type:	Original research
Date Submitted by the Author:	26-Feb-2021
Complete List of Authors:	Lundin, Susanne; Karolinska Institute Department of Clinical Science and Education Södersjukhuset; Sachs' Children's Hospital<br>Jonsson, M.; Karolinska Institute Department of Clinical Science and Education Södersjukhuset; Centre for Occupational and Environmental Medicine, Stockholm County Council<br>Wahlgren, Carl-Fredrik; Karolinska Institute Department of Medicine Solna, Dermatology Unit<br>Johansson, Emma; Karolinska Institute Department of Medicine Solna, Dermatology Unit.; Karolinska Universitetssjukhuset i Solna, Dermatology, Theme Inflammation and Infection<br>Bergstrom, Anna; Centre for Occupational and Environmental Medicine, Stockholm County Council; Karolinska Institute Institute of Environmental Medicine<br>Kull, Inger; Karolinska Institute Department of Clinical Science and Education Södersjukhuset, Karolinska Institutet; Sachs' Children's Hospital
**Primary Subject Heading**:	Dermatology
Secondary Subject Heading:	Dermatology, Qualitative research
Keywords:	Eczema < DERMATOLOGY, QUALITATIVE RESEARCH, Adult dermatology < DERMATOLOGY

I, the Submitting Author has the right to grant and does grant on behalf of all authors of the Work (as defined in the below author licence), an exclusive licence and/or a non-exclusive licence for contributions from authors who are: i) UK Crown employees; ii) where BMJ has agreed a CC-BY licence shall apply, and/or iii) in accordance with the terms applicable for US Federal Government officers or employees acting as part of their official duties; on a worldwide, perpetual, irrevocable, royalty-free basis to BMJ Publishing Group Ltd ("BMJ") its licensees and where the relevant Journal is co-owned by BMJ to the co-owners of the Journal, to publish the Work in this journal and any other BMJ products and to exploit all rights, as set out in our licence.

The Submitting Author accepts and understands that any supply made under these terms is made by BMJ to the Submitting Author unless you are acting as an employee on behalf of your employer or a postgraduate student of an affiliated institution which is paying any applicable article publishing charge ("APC") for Open Access articles. Where the Submitting Author wishes to make the Work available on an Open Access basis (and intends to pay the relevant APC), the terms of reuse of such Open Access shall be governed by a Creative Commons licence – details of these licences and which Creative Commons licence will apply to this Work are set out in our licence referred to above.

Other than as permitted in any relevant BMJ Author's Self Archiving Policies, I confirm this Work has not been accepted for publication elsewhere, is not being considered for publication elsewhere and does not duplicate material already published. I confirm all authors consent to publication of this Work and authorise the granting of this licence.

For peer review only

Young adults' perceptions of living with atopic dermatitis in relation to the concept of self-management - a qualitative study.

Short title: Living with eczema in relation to self-management.

Authors: *Susanne Lundin, PhD-student, RN* [a, b], *Marina Jonsson, PhD* [a, c, f], *Carl-Fredrik Wahlgren, MD, PhD, Professor* [d], *Emma Johansson, MD, PhD* [d, e], *Anna Bergström, PhD* [c, f] *& Inger Kull, Professor, PhD* [a, b]

Affiliations:

[a] Department of Clinical Science and Education, Södersjukhuset, Karolinska Institutet, SE-118 67 Stockholm, Sweden.

[b] Sachs' Children and Youth Hospital, Södersjukhuset, SE-118 83 Stockholm, Sweden.

[c] Centre of Occupational and Environmental Medicine, Stockholm County Council, SE-113 65 Stockholm, Sweden.

[d] Dermatology Unit, Department of Medicine, Solna, Karolinska Institutet, SE-171 77 Solna, Sweden.

[e] Dermatology, Theme Inflammation and Infection, Karolinska University Hospital, SE-171 76 Stockholm, Sweden.

[f] Institute of Environmental Medicine, Karolinska Institutet, SE-171 77 Solna, Sweden.

Corresponding Author: Susanne.lundin@ki.se

Susanne Lundin, Department of Clinical Science and Education, Södersjukhuset, Karolinska Institutet, Jägargatan 20, SE-118 67 Stockholm, Sweden, Tel: +46 707 730 124.

Acknowledgement: We thank all the participants in the BAMSE cohort, and all the staff involved in the study through the years.

Patient consent: Not required. All participants were orally and in writing informed that the results of the study would be published in a scientific journal. Details has been removed from case description to ensure anonymity.

Word Count: 3905/4000

For peer review only - http://bmjopen.bmj.com/site/about/guidelines.xhtml

Abstract

Objectives:

Learning to take control of one's health is an important part of the transition from adolescence to adulthood. This study aimed to explore young adults' perceptions of living with atopic dermatitis (AD) in relation to the concept of self-management.

Design:

A qualitative study with an inductive approach was performed through semi-structured interviews (n=15). The interviews were recorded, transcribed verbatim and analysed with systematic text condensation.

Participants:

Young adults (mean age 23,4 y) with persistent atopic dermatitis in a longitudinal population-based birth cohort. To capture experience of living with persistent AD (preschool/school-age onset) of different severity (mild to severe/very severe), a purposive selection was performed. In total 15 young adults were included. Persistent AD (preschool/school-age onset) was defined as dry skin in combination with itchy rash of typical localisation in the 12 months preceding the 16-year and the 24-year follow-ups. Severity was self-assessed using the Patient Oriented Eczema Measure.

Results:

Despite having experience of AD since childhood, the respondents expressed uncertainty about treatment and how it affected their bodies. Their uncertainties and feelings affected how they used topical corticosteroids. The respondents emphasised that they perceived availability of healthcare and knowledge about treatment of AD among healthcare providers to be limited.

For peer review only

The participants did not state any experiences of support to self-management from healthcare, which affect young adults' possibilities to take full control of their AD care.

Conclusions:

Young adults with preschool/school-age onset of AD are unsure how to treat and manage the disease. One explanation may be insufficient transition process.

Key words: Atopic dermatitis, Interviews, Self-management, Transition, Young adults

Word count, 250/300

For peer review only

For peer review only - http://bmjopen.bmj.com/site/about/guidelines.xhtml

Article summary

Strength and limitation

- Data from a longitudinal population-based birth cohort, with the possibility to obtaining detailed information about different phenotypes and severity of AD over time.
- The AD severity ranged from mild to severe/very severe as, measured by POEM, a validated questionnaire.
- Equal representation of young men and women.
- The results may not be transferred to young adults with more severe atopic dermatitis requiring specialist care.

For peer review only

Introduction

To optimise self-management in young people with chronic disease, there is a need for a transition process to increase knowledge and to achieve self-care skills and independence [1, 2]. To aid young adults to be autonomous and minimizing dependency from parents and healthcare regarding self-management of eczema, it is important that the transition process starts at an early age (11–13 years) in collaboration with the family and all stakeholders [3]. Atopic dermatitis (AD) [4] is a chronic disease that cannot be cured as yet and requires daily treatment [5]. In addition, it is known that eczema affects health-related quality of life (HRQoL) in both children and adults [6, 7]. The symptoms range from mild to very severe [8]. Most individuals with AD have a mild disease [6, 9].However. there seems to be a discordance between patients' and physicians' assessments of severity, judged using different instruments [10, 11]. The patients reported AD as more severe when their quality of life was impaired, while the physicians reported AD as more severe if sleep was reported to be disturbed [6, 7]. To ensure self-management in AD, it is important that the individuals have sufficient skills related to treatment principles and are aware of the goals of treatment, which are to increase disease control and to extend asymptomatic periods as much as possible [6, 12]. This requires knowledge and education. The mainstay of treatment is use of emollients every day to decrease dryness and extend time to relapse of AD [8]. The first-line treatment of inflammation consists of topical corticosteroids (TCSs) with differing potency depending on age, localisation, and severity of the symptoms [12]. The chronic, recurrent nature of the disease, [6] and the often-time-consuming treatment, to be performed every day, can be a challenge for individuals with AD [13]. Since parents have the main responsibility for the treatment of AD in their children, it is of importance that the parents have been trained to and understand the best management of the disease [8].A shift in responsibility takes place when the children reach adolescence, which requires that the adolescents gradually develop knowledge of their treatment. However,

For peer review only - http://bmjopen.bmj.com/site/about/guidelines.xhtml

results from a Swedish population-based birth cohort shows that many adolescents with AD are untreated [14].

Sex	Self-assessed severity at 16 y [1]	Self-assessed severity at 24 y [1]	Onset of eczema up to 4 years [2]	Onset of eczema from 8 years [3]

In Sweden, special transition models have been developed for chronic diseases such as congenital heart diseases and diabetes (still under evaluation) [15, 16] but – to the best of our knowledge – not for young persons with AD. According to the recently published guidelines on effective transition by the European Academy of Allergy and Clinical Immunology [3], learning to take control of treatment is an important part of the transition from adolescence to adulthood. Overall experiences of how young adults (from the general population) with AD manage the disease are scarcely studied. The aim of this qualitative study was therefore to explore young adults' perceptions of living with AD in relation to the concept of self-management.

Methods

Design

A qualitative semi-structured interview study with an inductive approach was used to capture young adults' experiences of AD in the concept of self-management. We followed the Consolidated Criteria for Reporting Qualitative Research guideline for conducting and reporting qualitative research [17]

Setting

The present study is a part of the longitudinal population-based cohort BAMSE [18, 19]. A purposive selection was performed to capture young adults with experience of persistent AD (preschool/school-age onset), with various severity (mild to severe/very severe) (Table 1).

For peer review only

For peer review only - http://bmjopen.bmj.com/site/about/guidelines.xhtml

M	Mild	Moderate		X
F	Mild	Moderate	X	
M	Moderate	Moderate	X	
M	Mild	Moderate	X	
M	Mild	Mild		X
F	Mild	Mild		X
M	Moderate	Severe/very severe	X	
M	Moderate	Mild	X	
F	Mild	Moderate	X	
F	Mild	Severe/very severe	X	
F	Severe/very severe	Moderate	X	
M	Mild	Severe/very severe	X	
F	Moderate	Moderate	X	
F	Mild	Severe/very severe		X
M	Severe/very severe	Severe/very severe	X	

Table 1. Clinical description of sex, severity, and onset of AD among the participants.

[1] Patient Oriented Eczema Measure class 1, mild eczema (3–7), class 2, moderate eczema (8–16), class 3, severe/very severe eczema (17–28).
[2] Preschool eczema (i.e., eczema onset at 1,2 or 4, not 8, 12 or 16 years of age)
[3] School age eczema (i.e., eczema onset at 8, 12 or 16 not at 1,2 or 4 years of age)

Moreover, we strived to capture an equal proportion of men and women with different severity of AD. They were invited to the interview study by a clinical research nurse over the telephone. Thereafter, the first author contacted the participants, informed them about the study, and asked if they wanted to share their experiences of living with AD.

Inclusion criteria

For peer review only

Young adults with persistent AD, defined as dry skin in combination with itchy rash of typical localisation (neck/throat, arm/leg flexures and/or wrists/ankles) in the 12 months preceding the 16- and the 24-year follow-ups. The severity of AD was self-assessed as mild to severe/very severe using the Patient Oriented Eczema Measure (POEM) [20], which enabled us to capture a range of experiences. The young adults who fulfilled both the questionnaire definition of AD (as above) and the clinical definition of AD; visible AD on the neck/throat and/or flexure surfaces of the arms/legs and/or wrists/ankles and/or reported symptoms of

**Themes related to experiences of eczema, treatment and care**	**Follow-up questions**
Tell me what it is like to live with eczema.	Tell me what it has been like lately.<br>What was it like when you were a teenager?<br>Is there any difference?
Tell me what it is like when you have trouble with your eczema.	What is it like when your eczema is not troubling you?<br>How often do you have trouble with your eczema?<br>What do you think is the cause of your eczema?
Tell me how you usually self-treat.	How do you feel it works?<br>How has treatment been shown or explained to you?<br>What do you think about cortisone?

itchy skin in the preceding week as assessed by a trained nurse were included.

Data collection

All interviews were conducted at the research clinic, except two, which were conducted by telephone. The interviews were performed by the first author, an experienced dermatology nurse (SL) with no connection to the study participants before the interviews. The semi-structured interview guide consisted of five themes related to living with AD: daily life, symptoms, treatment, care, and consequences of the disease (Table 2).

Table 2. Interview guide.

For peer review only

	What do you think about moisturizing cream?
Tell me about the experiences you have of healthcare in relation to your eczema.	Tell me about the needs you have from healthcare due to your eczema.<br>Tell me about any information you have received from healthcare regarding your eczema.<br>Could it have been given in any other way?
In what situations do you think about your eczema?	Do you avoid doing anything because of your eczema?<br>Have you avoided anything in the past because of your eczema?

Probing questions were used to get a deeper understanding or explanation of some statements. To test the interview guide, one pilot interview was conducted (not included). Minor changes were then made to the interview guide. During the interview, the participants were asked if their answers were interpreted correctly. Thus, the interviewer received confirmation from the respondents. The interviews lasted on average 31 (range 23–43) minutes. Data collection continued until information power was judged to have been achieved. This is an approach which considers the aim, the sample specificity, the theoretical background, the quality of dialogue and the strategy for analyses. Taken together, these determine whether sufficient information power has been obtained. In total, 15 young adults, mean age 23,4 y were included (Table 1), which gave us rich data covering the purpose of the study. All interviews were recorded and transcribed verbatim.

Data analysis

The text was analysed by three researchers (SL, MJ & IK) in collaboration, using systematic text condensation (STC), a thematic cross-case strategy in four steps [21]. First, all interviews were read through (SL, MJ & IK), and preliminary themes were identified based on the contents of the text (SL & MJ). In the second step, meanings units were identified based on the contents of the preliminary themes and code groups were developed (SL & MJ). In the

For peer review only - http://bmjopen.bmj.com/site/about/guidelines.xhtml

third step, the material from each code group was decontextualized, sorted into subgroups and the contents of the text were condensed (SL, MJ & IK). Lastly, in the fourth step, data were reconceptualised, and categories made based on the essence of the young adults' experiences of eczema (SL, MJ & IK) (Table 3).

Table 3. The process of analysing young adults' experiences of living with AD with systematic text condensation.

Step 1.<br>Preliminary themes	Step2.<br>Codes	Step 3.<br>Subgroups	Step 4.<br>Categories
Reminds you	*Always on my mind*<br>*Almost never symptom-free*<br>*As long as it is not visible*	*Strategies*<br>*Acceptance*	*A part of life*
The care is vague	*They do not have time*<br>*Everyone says different things*	*Limited availability*<br>*Limited support*	*Difficult to get help*
	Lack of information<br>*The treatment does not work*	*Uncertainty*<br>*Need for information*	*Limited knowledge*
Physical effects	*Not good for my body*<br>*Being careful*<br>*Burning and stinging*	*Unnatural*<br>*It hurts*	*Impact on my body*

The analysis was inductive, without any theory driven approach to the phenomenon of self-management. We were open to the data and what the material told us, from an individual to a general level. In the analysis process we tried to bracket our preconceptions to minimize the impact of our professional and clinical experiences. Using STC, with many similarities to the editing analysis style [22] we developed, categories derived from the empirical data.

For peer review only - http://bmjopen.bmj.com/site/about/guidelines.xhtml

Ethics

The study was approved by the regional ethical review board at Karolinska Institutet, Stockholm (approval number 2016/1380-31-2, 2017/395-32). All participants gave informed consent, 13 with written consent and two by telephone.

Patient and public involvement

The primary intention of this study was to capture young adults' perceptions of living with AD in relation to self-management. However, the young adults were not involved in the design of the study.

Results

The respondents' perceptions of living with AD in the concept of self-management resulted in four categories and eight subgroups (Table 3). Despite having had AD since childhood, the respondents expressed uncertainty about treatment and how it could affect their bodies. Moreover, both symptoms and treatment were described as painful. Their uncertainties and feelings affected how they used TCSs. Though the disease made itself known all the time, most of the participants stated that they had gotten used to living with eczema, it was a part of their life. The respondents emphasised that they experienced that both the accessibility and the support from healthcare providers were limited.

Limited knowledge

Although most of the respondents had had AD from an early age, they expressed that their knowledge about its cause and treatment was limited. They had created their own treatment

For peer review only

For peer review only - http://bmjopen.bmj.com/site/about/guidelines.xhtml

routines, based on their experiences and/or what they have learned from their parents. The young adults stated that they experienced a lack of information and directives on treatment.

Uncertainty

The young adults expressed uncertainty about the cause of their AD, how they should treat it, what they should use to treat it, where on the body, and for how long. They had not received much information about the cause or variability of the disease, or about different treatments.

"The doctors have been more like it will go away when you reach a certain age or something, it's just now, it will disappear, so ... I'm just waiting for that age to come" (9)

They felt uncertain about the treatment, if they were doing the right thing, and if the topical treatment should include or consist of cortisone.

"They say cortisone is what helps, I don't know, there's no cortisone in Mometasone, as far as I know" (3)

Need for information.

There was a general belief among respondents that TCSs attenuated or diminished symptoms only for the moment, which reinforced the belief that they could have "become immune" to these creams.

Most of the young adults said they treated themselves regularly, but it was clear they were primarily referring to moisturising creams. The respondents mentioned different treatment recommendations they had been given over the years, such as not to use TCSs around the eyes and not to apply cream if it was cold outside. They also reported that they were advised to use TCSs sparingly, at most twice a week, without regard to the severity of the AD.

For peer review only - http://bmjopen.bmj.com/site/about/guidelines.xhtml

The respondents expressed that it would be helpful with more information and requested a guide with treatment principles, depending on the severity of the AD. This would be important in order to achieve independence regarding self-management of the disease.

"I have understood by, like, reading and talking with different people that you should have different, like, cycles for it, but I haven't really understood how to do it, because I have not got that input from a doctor, how I should do it" (14)

Impact on my body

Both symptoms and treatment of the AD had a painful impact on the body. Moreover, the need to use different creams to treat the AD was something that felt unnatural and there were thoughts on various treatments adversely affecting the body.

It hurts

Many of the respondents stated that one of the most difficult things about AD was that it hurt. They described the pain as a burning and stinging feeling under the skin. That pain was annoying and could prevent them from doing things that they liked to do. The pain was sometimes more trying than the itching.

"Periodically, it hurt too much to put on socks and shoes…" (1)

They stated that it was painful to apply cream when the AD was severe; they self-treated to get rid of the pain, but that hurt even more.

"It stings, of course, every time I apply the cream" (15)

Unnatural

For peer review only - http://bmjopen.bmj.com/site/about/guidelines.xhtml

Some of the respondents felt that it was unnatural to cover their skin with something that could affect the body negatively, especially in the case of TCSs.

"That you absorb, like, products that the body is not really made to deal with" (2)

They had heard that the skin could become damaged and thinner from using TCSs, which made them anxious; they therefore used these as little as possible. They felt that it was strange to use a treatment that could simultaneously damage the skin even if it decreased the AD.

"I try not to use too much, plain and simple, and hope it is…that I'm like doing what you're supposed to" (7)

Difficult to get help.

There was a perception among the young adults that it was difficult to get help from healthcare, both making an appointment and getting the "right" treatment. The respondents did not state any experiences of education in self-management by healthcare. Moreover, they perceived that healthcare providers did not recognise the symptoms, which resulted in insufficient and delayed treatment.

Limited availability

Even though most of the respondents had had AD all their life, they stated that they had limited contact with healthcare. It seemed to be difficult to get in touch with healthcare and get appropriate help, even when the AD was severe. Sometimes, it took several months to get an appointment and they needed to spend a lot of time trying to get in touch with the care provider. Many of the respondents could not remember the last time they had had an appointment.

"It was probably in August that I contacted my dermatology department, because it [i.e., eczema] was so very bad…and then I got an appointment for the middle of November" (14)

For peer review only - http://bmjopen.bmj.com/site/about/guidelines.xhtml

Limited support

One respondent had experienced that the symptoms of the AD had not been taken seriously; the physician downplayed the respondent's symptoms and compared them with the more widespread symptoms of others. The participants perceived that physicians in general had limited knowledge about treatment of AD and the only advice they had received was to apply topical creams, without further instructions.

"It seems that the GP doesn't know all that much about eczema…and just prescribes ointments that do not help" (8)

Information about how they should treat the eczema also differed from physician to physician.

"Some of them say 'apply it when you need to,' others say, 'apply it like several times a day,' it's like people are guessing a little, they don't have any idea, really" (10)

A part of life

The respondents stated that they had become accustomed to living with AD and had accepted their disease. Even so, they had many strategies about how they should avoid worsening of the AD, which required a lot of routines in the form of regular treatment, avoiding scratching, and creating strategies in daily life.

Strategies

Most of the respondents thought of and were aware of their AD constantly: when it was itching, when it was visible, when they had to apply cream, and so on. They had developed different strategies to avoid worsening of the AD. The respondents perceived it as trying to experience itchiness all the time and they were aware of the importance of not scratching, even though it was difficult. Trying not to scratch required constant vigilance. Scratching was

For peer review only - http://bmjopen.bmj.com/site/about/guidelines.xhtml

the only thing they wanted to do, but they knew it would immediately result in them being "punished", through worsening of the AD.

"That's the main thing in how I handle it, that just sitting there thinking that okay, don't scratch, don't scratch, don't scratch, don't scratch, don't scratch, it has become like a habit to try not to do it" (15)

The major problem was that the AD never disappeared. They had to make choices in their daily life based on their skin condition. Most of the respondents never felt free of symptoms, which they found trying.

"It's probably all the time I'd say, on different parts, it's like it moves around a bit, where it's, like, worst" (10)

The treatment of AD was demanding, according to the respondents. Treatment was a bit awkward and nothing they enjoyed. They had to have a regular routine and a strategy; even then, it took a long time to get rid of the AD and the respondents stated that the creams only helped for the moment.

"But the eczema keeps, like, coming back anyway, even though I have this routine" (9)

They always had to carry creams with them, to school, work, or when they travelled, and if they forgot they were "punished" at once, through worsening of the AD.

"Always having creams with you and applying them is not all that much fun either" (3)

Acceptance

The respondents emphasised that they had gotten used to living with AD and had accepted their situation, even though it was hard. They did not allow AD to keep them from doing things. It was a part of their life.

For peer review only - http://bmjopen.bmj.com/site/about/guidelines.xhtml

"It's, like, something you have, you have to learn to live with it, maybe" (7)

While they had accepted their disease, they tried to avoid revealing their AD, in so far as possible, when it was in visible places on their bodies.

"I do not like it to be visible, so usually when I have these red, I usually use long sleeves, to like cover up" (13)

Discussion

This qualitative interview study of young adults with persistent AD from the general population demonstrated several shortcomings in the concept of self-management. Even though the young adults had experience of persistent AD, they were unsure about how to treat the AD. The respondents expressed limited knowledge about the differences between emollients and TCSs and regarding the potency of the latter. Their uncertainty may be related to their perception of difficulties in getting help from healthcare in the terms of limited knowledge and lack of support about treatment of AD among healthcare providers. While acceptance of the disease was high, the respondents never felt completely symptom-free. Altogether, this indicates an insufficiency regarding self-management, including knowledge and skills in treatment principles for the disease, which may affect young adults' possibilities to take full control of their AD care.

Knowledge about treatment of eczema

The young adults' uncertainty regarding how to treat and manage the AD was in accordance with other studies. The uncertainty seems to be found also in self-treating adults, even if their

For peer review only - http://bmjopen.bmj.com/site/about/guidelines.xhtml

AD is more severe; this can delay the start of treatment in flare-ups [23]. In contrast, adolescents (12-18 y) in a qualitative study [24] expressed satisfaction with the effect of their treatments, even though half of them used TCSs every day because of low efficiency. Moreover, they had limited knowledge and incorrect beliefs about how the treatment worked. The same beliefs were seen in the present study, but the participants in our study expressed less satisfaction and more often believed that treatment, even with emollients, could harm their body.

For peer review only

For peer review only - http://bmjopen.bmj.com/site/about/guidelines.xhtml

Factors of importance for self-management

The limited contact with and difficulties in getting help from healthcare can to some extent be due to limited knowledge regarding whom to contact. Healthcare in Sweden is divided into primary care, specialist care, and highly specialised care [25]. Primary care is responsible for treatment and care of the most common skin diseases, such as AD. Individuals with AD can buy up to 30 g of mild TCSs over the counter from a pharmacy; for larger amounts or more potent TGCs, a prescription is needed. The respondents' opinions that physicians had limited knowledge about treatment is in accordance with the results from a qualitative study including GPs. The GPs stated that they used a trial-and-error approach when prescribing emollients, due to the large assortment of different creams, and that they were reluctant to prescribe strong TCSs to children [26]. The prescribed amounts of emollients in primary care have reportedly been unexpectedly low when compared with guidelines, which was also seen for prescription of TCSs in specialist care [27, 28]. To have the possibility to treat AD adequately, the individuals need to have access to appropriate potency and amount of TCSs and to treat the AD daily until the symptoms clear. Thereafter, the TCS therapy should be changed into proactive treatment twice a week [8].

Concerns about side-effects of TCSs are common. Studies including both children and adults with moderate to severe AD have found that approximately 50% of guardians/patients are reluctant to use TCSs [29, 30]. In addition, misleading information from relatives/friends, the internet, or pharmacies can lead to poor adherence [31, 32].

Moreover, it has been shown that concerns and uncertainties about side-effects exist even among healthcare providers [33]. Both GPs and pharmacists inform patients about the risks of TCSs and that they should be applied sparingly and not over long periods of time [34]. This can

For peer review only - http://bmjopen.bmj.com/site/about/guidelines.xhtml

affect the potency and amount of TCSs prescribed and strengthen the feeling among individuals with AD that a TCS is something to be careful with [35, 36].

While the young adults in the present study had gotten used to living with AD, they stated that they needed more support. Furthermore, there seemed to be a need for repeated information about the cause, course, and treatment of the disease. Several studies have stated the importance of shared decision-making, education, and a written action plan [37-40], which could be useful tools to achieve independence. This is important in the process of developing autonomy in self-management and has been confirmed in a study among youths aged 14–24 years [41], which showed that 41% wanted to be more involved in their care. For adolescents with asthma, it is known that support during the transition process results in better adherence to treatment, but the healthcare transition in AD is less studied [42]. Guidelines on asthma emphasise the importance of reviewing each patient's inhalation technique at every healthcare visit, to decrease the risk of exacerbations [43]. It would be equally important to review how patients with AD perform self-treatment, to achieve as long symptom-free periods as possible.

Strengths and limitations

One strength of the present study was that all respondents had well-defined AD based on both prospectively recorded questionnaire data and clinical examinations. Moreover, their AD severity ranged from mild to very severe. Additional strengths are the credibility and transferability based on the wide range of data from the respondents, which we have exemplified using the respondents' own words.

The respondents were from the general population, which is a strength since most patients with AD are treated in primary care. However, the results may not be transferred to patients

For peer review only - http://bmjopen.bmj.com/site/about/guidelines.xhtml

with more severe AD, who may have more contact with specialist care. Still, some of the respondents in the present study had moderate to very severe AD.

In qualitative studies, the number of participants is generally low. However, this can be compensated by a purposive selection, to get a deeper understanding of the respondents' experiences. We recruited participants with long experience of mild to very severe AD, who wanted to share their experiences. The data collection gave us varied and rich data on perceptions and experiences of living with AD. An interviewer's preconception about subjects can be both positive and negative. Deep experience can be helpful in the interview situation to promote understanding and when asking probing questions, but can also be negative, leading to assumed understanding of what informants are saying and an assumption that the interviewer and respondent are thinking about the same thing. Preconceptions can also be negative during the analysis phase and the bracketing of the preconceptions is a way to try to avoid these influences. Even so this may be an unattainable goal according to Malterud [44]. To minimise these effects, the analysis was carried out in collaboration with two co-authors with little or no experience in the care of patients with AD.

Conclusions

This qualitative interview study of young adults with AD from the general population demonstrated several shortcomings in self-management. Despite long experience of AD, the young adults expressed limited knowledge and uncertainty about treatment. Their uncertainty may be related to their perception of difficulties in getting help from healthcare and of limited support, including knowledge about treatment of AD among healthcare providers. Altogether this may indicate shortcomings in the transition process.

For peer review only - http://bmjopen.bmj.com/site/about/guidelines.xhtml

Authors Contributions: SL, MJ and IK contributed to conception and design of the study. The interviews were managed by SL. The analysis with Systematic Text condensation was conducted by SL, MJ and IK in collaboration. Drafting of the manuscript were conducted by SL with support of IK, C-F. W, EJ and AB. All authors participated in critical revision of the manuscript, provided important intellectual input, and approved the final version.

Conflict of interest: E. Johansson reports personal lecture fees from Sanofi-Genzyme, LEO Pharma, Novartis, and ACO. E. Johansson and S. Lundin has been part of the advisory board at Sanofi-Genzyme. The rest of the authors declare no relevant conflict of interest.

Funding: Funding for this study was provided by the Swedish Asthma and Allergy Association, grant/award number 2017-0007. The funder has not been involved in the design and conduct of the study; collection, management, analysis, and interpretation of the data; preparation, review, or approval of the manuscript; and decision to submit the manuscript for publication.

Data sharing statement: No data are available. Audio files and transcribed text has been deposited anonymously at the BAMSE-secretariat, Institute of Environmental Medicine at Karolinska Institutet, Stockholm, Sweden.

References

1. Gabriel P, McManus M, Rogers K, White P. Outcome Evidence for Structured Pediatric to Adult Health Care Transition Interventions: A Systematic Review. *The Journal of pediatrics*. Sep 2017;188:263-269.e15. doi:10.1016/j.jpeds.2017.05.066
2. Schmidt A, Ilango SM, McManus MA, Rogers KK, White PH. Outcomes of Pediatric to Adult Health Care Transition Interventions: An Updated Systematic Review. *Journal of pediatric nursing*. Mar-Apr 2020;51:92-107. doi:10.1016/j.pedn.2020.01.002
3. Roberts G, Vazquez-Ortiz M, Knibb R, et al. EAACI Guideline on the effective transition of adolescents and young adults with allergy and asthma. *Allergy*. Jun 19 2020;doi:10.1111/all.14459
4. Kantor R, Thyssen JP, Paller AS, Silverberg JI. Atopic dermatitis, atopic eczema, or eczema? A systematic review, meta-analysis, and recommendation for uniform use of 'atopic dermatitis'. *Allergy*. Oct 2016;71(10):1480-5. doi:10.1111/all.12982
5. Bieber T. Atopic dermatitis. *Annals of dermatology*. May 2010;22(2):125-37. doi:10.5021/ad.2010.22.2.125
6. Weidinger S, Novak N. Atopic dermatitis. *Lancet*. Mar 12 2016;387(10023):1109-22. doi:10.1016/S0140-6736(15)00149-X
7. Grant L, Seiding Larsen L, Trennery C, et al. Conceptual Model to Illustrate the Symptom Experience and Humanistic Burden Associated With Atopic Dermatitis in Adults and Adolescents. *Dermatitis : contact, atopic, occupational, drug*. Jul/Aug 2019;30(4):247-254. doi:10.1097/der.0000000000000486
8. Wollenberg A, Barbarot S, Bieber T, et al. Consensus-based European guidelines for treatment of atopic eczema (atopic dermatitis) in adults and children: part I. *Journal of the European Academy of Dermatology and Venereology : JEADV*. May 2018;32(5):657-682. doi:10.1111/jdv.14891

For peer review only - http://bmjopen.bmj.com/site/about/guidelines.xhtml

9. Ballardini N, Kull I, Soderhall C, Lilja G, Wickman M, Wahlgren CF. Eczema severity in preadolescent children and its relation to sex, filaggrin mutations, asthma, rhinitis, aggravating factors and topical treatment: a report from the BAMSE birth cohort. *Br J Dermatol*. Mar 2013;168(3):588-94. doi:10.1111/bjd.12196
10. Wei W, Anderson P, Gadkari A, et al. Discordance Between Physician- and Patient-Reported Disease Severity in Adults with Atopic Dermatitis: A US Cross-Sectional Survey. *American journal of clinical dermatology*. Dec 2017;18(6):825-835. doi:10.1007/s40257-017-0284-y
11. Holm EA, Wulf HC, Stegmann H, Jemec GB. Life quality assessment among patients with atopic eczema. *Br J Dermatol*. Apr 2006;154(4):719-25. doi:10.1111/j.1365-2133.2005.07050.x
12. Weidinger S, Beck LA, Bieber T, Kabashima K, Irvine AD. Atopic dermatitis. *Nature reviews Disease primers*. Jun 21 2018;4(1):1. doi:10.1038/s41572-018-0001-z
13. Santer M, Burgess H, Yardley L, et al. Managing childhood eczema: qualitative study exploring carers' experiences of barriers and facilitators to treatment adherence. *Journal of advanced nursing*. Nov 2013;69(11):2493-501. doi:10.1111/jan.12133
14. Lundin S, Wahlgren CF, Bergstrom A, et al. Use of emollients and topical glucocorticoids among adolescents with eczema - datafrom the population-based birth cohort BAMSE. *Br J Dermatol*. Feb 25 2018;doi:10.1111/bjd.16484
15. Acuña Mora M, Saarijärvi M, Sparud-Lundin C, Moons P, Bratt EL. Empowering Young Persons with Congenital Heart Disease: Using Intervention Mapping to Develop a Transition Program - The STEPSTONES Project. *Journal of pediatric nursing*. Jan-Feb 2020;50:e8-e17. doi:10.1016/j.pedn.2019.09.021
16. Brorsson AL, Bratt EL, Moons P, et al. Randomised controlled trial of a person-centred transition programme for adolescents with type 1 diabetes (STEPSTONES-DIAB): a study protocol. *BMJ open*. Apr 14 2020;10(4):e036496. doi:10.1136/bmjopen-2019-036496
17. Tong A, Sainsbury P, Craig J. Consolidated criteria for reporting qualitative research (COREQ): a 32-item checklist for interviews and focus groups. *International journal for quality in health care : journal of the International Society for Quality in Health Care*. Dec 2007;19(6):349-57. doi:10.1093/intqhc/mzm042
18. Wickman M, Kull I, Pershagen G, Nordvall SL. The BAMSE project: presentation of a prospective longitudinal birth cohort study. *Pediatr Allergy Immunol*. 2002;13 Suppl 15:11-3.
19. Ödling M, Andersson N, Hallberg J, et al. A Gap Between Asthma Guidelines and Management for Adolescents and Young Adults. *The journal of allergy and clinical immunology In practice*. Oct 2020;8(9):3056-3065.e2. doi:10.1016/j.jaip.2020.05.034
20. Charman CR, Venn AJ, Williams HC. The patient-oriented eczema measure: development and initial validation of a new tool for measuring atopic eczema severity from the patients' perspective. *Arch Dermatol*. Dec 2004;140(12):1513-9. doi:10.1001/archderm.140.12.1513
21. Malterud K. Systematic text condensation: a strategy for qualitative analysis. *Scandinavian journal of public health*. Dec 2012;40(8):795-805. doi:10.1177/1403494812465030
22. Crabtree BF, Miller WL. *Doing qualitative research*. SAGE; 1999.
23. Ortiz de Frutos FJ, Torrelo A, de Lucas R, et al. Patient perspectives on triggers, adherence to medical recommendations, and disease control in atopic dermatitis: the DATOP study. *Actas dermo-sifiliograficas*. Jun 2014;105(5):487-96. doi:10.1016/j.ad.2014.01.004
24. Kosse RC, Bouvy ML, Daanen M, de Vries TW, Koster ES. Adolescents' Perspectives on Atopic Dermatitis Treatment-Experiences, Preferences, and Beliefs. *JAMA Dermatol*. Jul 1 2018;154(7):824-827. doi:10.1001/jamadermatol.2018.1096
25. Council SR. The Swedish healthcare system. Updated 2020-02-25. Accessed 2020-07-01,
26. Le Roux E, Powell K, Banks JP, Ridd MJ. GPs' experiences of diagnosing and managing childhood eczema: a qualitative study in primary care. *The British journal of general practice : the journal of the Royal College of General Practitioners*. Feb 2018;68(667):e73-e80. doi:10.3399/bjgp18X694529
27. Choi JY, Dawe R, Ibbotson S, Fleming C, Doney A, Foerster J. Topical treatments in atopic dermatitis: unexpectedly low use of emollients; use of topical corticosteroid is higher in juvenile

For peer review only - http://bmjopen.bmj.com/site/about/guidelines.xhtml

patients, higher in male vs females, and shows independent associations with asthma and depression. *Br J Dermatol*. Jun 30 2019;doi:10.1111/bjd.18265
28. Schmitt J, Schmitt NM, Kirch W, Meurer M. Outpatient care and medical treatment of children and adults with atopic eczema. *Journal der Deutschen Dermatologischen Gesellschaft = Journal of the German Society of Dermatology : JDDG*. Apr 2009;7(4):345-51. doi:10.1111/j.1610-0387.2008.06967.x
29. Zuberbier T, Orlow SJ, Paller AS, et al. Patient perspectives on the management of atopic dermatitis. *J Allergy Clin Immunol*. Jul 2006;118(1):226-32. doi:10.1016/j.jaci.2006.02.031
30. Jung HJ, Bae JY, Kim JE, et al. Survey of disease awareness, treatment behavior and treatment satisfaction in patients with atopic dermatitis in Korea: A multicenter study. *The Journal of dermatology*. Oct 2018;45(10):1172-1180. doi:10.1111/1346-8138.14540
31. Smith SD, Farrugia LL, Harris V, et al. Evaluation of the influence of family and friends, and the Internet on patient perceptions of long-term topical corticosteroid use. *The Journal of dermatological treatment*. Nov 2017;28(7):642-646. doi:10.1080/09546634.2017.1306017
32. Smith SD, Hong E, Fearns S, Blaszczynski A, Fischer G. Corticosteroid phobia and other confounders in the treatment of childhood atopic dermatitis explored using parent focus groups. *The Australasian journal of dermatology*. Aug 2010;51(3):168-74. doi:10.1111/j.1440-0960.2010.00636.x
33. Bos B, Antonescu I, Osinga H, Veenje S, de Jong K, de Vries TW. Corticosteroid phobia (corticophobia) in parents of young children with atopic dermatitis and their health care providers. *Pediatric dermatology*. Jan 2019;36(1):100-104. doi:10.1111/pde.13698
34. Farrugia LL, Lee A, Fischer G, Blaszczynski A, Carter SR, Smith SD. Evaluation of the influence of pharmacists and GPs on patient perceptions of long-term topical corticosteroid use. *The Journal of dermatological treatment*. Mar 2017;28(2):112-118. doi:10.1080/09546634.2016.1213353
35. Arkwright PD, Motala C, Subramanian H, Spergel J, Schneider LC, Wollenberg A. Management of difficult-to-treat atopic dermatitis. *The journal of allergy and clinical immunology In practice*. Mar 2013;1(2):142-51. doi:10.1016/j.jaip.2012.09.002
36. Feldman SR, Vrijens B, Gieler U, Piaserico S, Puig L, van de Kerkhof P. Treatment Adherence Intervention Studies in Dermatology and Guidance on How to Support Adherence. *American journal of clinical dermatology*. Apr 2017;18(2):253-271. doi:10.1007/s40257-017-0253-5
37. Noerreslet M, Jemec GB, Traulsen JM. Involuntary autonomy: patients' perceptions of physicians, conventional medicines and risks in the management of atopic dermatitis. *Social science & medicine (1982)*. Nov 2009;69(9):1409-15. doi:10.1016/j.socscimed.2009.08.036
38. Stalder JF, Bernier C, Ball A, et al. Therapeutic patient education in atopic dermatitis: worldwide experiences. *Pediatric dermatology*. May-Jun 2013;30(3):329-34. doi:10.1111/pde.12024
39. Muller SM, Tomaschett D, Euler S, Vogt DR, Herzog L, Itin P. Topical Corticosteroid Concerns in Dermatological Outpatients: A Cross-Sectional and Interventional Study. *Dermatology (Basel, Switzerland)*. 2016;232(4):444-52. doi:10.1159/000446068
40. Shi VY, Nanda S, Lee K, Armstrong AW, Lio PA. Improving patient education with an eczema action plan: a randomized controlled trial. *JAMA Dermatol*. Apr 2013;149(4):481-3. doi:10.1001/jamadermatol.2013.2143
41. Schuiteman S, Chua KP, Plegue MA, Ilyas O, Chang T. Self-Management of Health Care Among Youth: Implications for Policies on Transitions of Care. *The Journal of adolescent health : official publication of the Society for Adolescent Medicine*. Feb 26 2020;doi:10.1016/j.jadohealth.2020.01.009
42. Vazquez-Ortiz M, Angier E, Blumchen K, et al. Understanding the challenges faced by adolescents and young adults with allergic conditions: a systematic review. *Allergy*. Mar 6 2020;doi:10.1111/all.14258
43. GINA gifa. Pocket guide for asthma management and prevention (for adults and children older than 5 years). Updated 2020-02-28.
44. Malterud K. *Kvalitativa metoder i medicinsk forskning : en introduktion (Qualitative methods in medical research: an introduction)*. Studentlitteratur; 2014.

For peer review only - http://bmjopen.bmj.com/site/about/guidelines.xhtml

Table 1

Consolidated criteria for reporting qualitative studies (COREQ): 32-item checklist

No	Item	Guide questions/description
**Domain 1: Research team and reflexivity**		
Personal Characteristics		
1.	Interviewer/facilitator	Which author/s conducted the interview or focus group? page 1
2.	Credentials	What were the researcher's credentials? *E.g. PhD, MD* page 1
3.	Occupation	What was their occupation at the time of the study? page 8
4.	Gender	Was the researcher male or female? page
5.	Experience and training	What experience or training did the researcher have? page 8
Relationship with participants		
6.	Relationship established	Was a relationship established prior to study commencement? No
7.	Participant knowledge of the interviewer	What did the participants know about the researcher? e.g. *personal goals, reasons for doing the research* At start of the interviews the author introduce herself as RN + PhD-Student

8.	Interviewer characteristics	What characteristics were reported about the interviewer/facilitator? e.g. *Bias, assumptions, reasons and interests in the research topic* page 8, 18
**Domain 2: study design**		
Theoretical framework		
9.	Methodological orientation and Theory	What methodological orientation was stated to underpin the study? *e.g. grounded theory, discourse analysis, ethnography, phenomenology, content analysis* page 9
Participant selection		
10.	Sampling	How were participants selected? *e.g. purposive, convenience, consecutive, snowball* page 7
11.	Method of approach	How were participants approached? e.g. *face-to-face, telephone, mail, email* page 7
12.	Sample size	How many participants were in the study? page 8
13.	Non-participation	How many people refused to participate or dropped out? Reasons? page 8
Setting		
14.	Setting of data collection	Where was the data collected? e.g. *home, clinic, workplace* page 8
15.	Presence of non-participants	Was anyone else present besides the participants and researchers? No

16.	Description of sample	What are the important characteristics of the sample? *e.g. demographic data, date*	page 8
Data collection			
17.	Interview guide	Were questions, prompts, guides provided by the authors? Was it pilot tested?	page 8
18.	Repeat interviews	Were repeat interviews carried out? If yes, how many?	No
19.	Audio/visual recording	Did the research use audio or visual recording to collect the data?	page 8
20.	Field notes	Were field notes made during and/or after the interview or focus group?	No
21.	Duration	What was the duration of the interviews or focus group?	page 8
22.	Data saturation	Was data saturation discussed?	page 8
23.	Transcripts returned	Were transcripts returned to participants for comment and/or correction?	No
**Domain 3: analysis and findings**z			
Data analysis			
24.	Number of data coders	How many data coders coded the data?	
25.	Description of the coding tree	Did authors provide a description of the coding tree?	page 8 + Figure 1+2

26.	Derivation of themes	Were themes identified in advance or derived from the data?	page 9
27.	Software	What software, if applicable, was used to manage the data?	–
28.	Participant checking	Did participants provide feedback on the findings?	No
Reporting			
29.	Quotations presented	Were participant quotations presented to illustrate the themes / findings? Was each quotation identified? *e.g. participant number*	page 10-15
30.	Data and findings consistent	Was there consistency between the data presented and the findings?	page 10-15
31.	Clarity of major themes	Were major themes clearly presented in the findings?	page 10-15
32.	Clarity of minor themes	Is there a description of diverse cases or discussion of minor themes?	–

BMJ Open

Young adults' perceptions of living with atopic dermatitis in relation to the concept of self-management - a qualitative study

Journal:	*BMJ Open*
Manuscript ID	bmjopen-2020-044777.R2
Article Type:	Original research
Date Submitted by the Author:	28-May-2021
Complete List of Authors:	Lundin, Susanne; Karolinska Institute Department of Clinical Science and Education Södersjukhuset; Sachs' Children's Hospital<br>Jonsson, M.; Karolinska Institute Department of Clinical Science and Education Södersjukhuset; Centre for Occupational and Environmental Medicine, Stockholm County Council<br>Wahlgren, Carl-Fredrik; Karolinska Institute Department of Medicine Solna, Dermatology Unit<br>Johansson, Emma; Karolinska Institute Department of Medicine Solna, Dermatology Unit.; Karolinska Universitetssjukhuset i Solna, Dermatology, Theme Inflammation and Infection<br>Bergstrom, Anna; Centre for Occupational and Environmental Medicine, Stockholm County Council; Karolinska Institute Institute of Environmental Medicine<br>Kull, Inger; Karolinska Institute Department of Clinical Science and Education Södersjukhuset, Karolinska Institutet; Sachs' Children's Hospital
**Primary Subject Heading**:	Dermatology
Secondary Subject Heading:	Dermatology, Qualitative research
Keywords:	Eczema < DERMATOLOGY, QUALITATIVE RESEARCH, Adult dermatology < DERMATOLOGY

SCHOLARONE™
Manuscripts

I, the Submitting Author has the right to grant and does grant on behalf of all authors of the Work (as defined in the below author licence), an exclusive licence and/or a non-exclusive licence for contributions from authors who are: i) UK Crown employees; ii) where BMJ has agreed a CC-BY licence shall apply, and/or iii) in accordance with the terms applicable for US Federal Government officers or employees acting as part of their official duties; on a worldwide, perpetual, irrevocable, royalty-free basis to BMJ Publishing Group Ltd ("BMJ") its licensees and where the relevant Journal is co-owned by BMJ to the co-owners of the Journal, to publish the Work in this journal and any other BMJ products and to exploit all rights, as set out in our licence.

The Submitting Author accepts and understands that any supply made under these terms is made by BMJ to the Submitting Author unless you are acting as an employee on behalf of your employer or a postgraduate student of an affiliated institution which is paying any applicable article publishing charge ("APC") for Open Access articles. Where the Submitting Author wishes to make the Work available on an Open Access basis (and intends to pay the relevant APC), the terms of reuse of such Open Access shall be governed by a Creative Commons licence – details of these licences and which Creative Commons licence will apply to this Work are set out in our licence referred to above.

Other than as permitted in any relevant BMJ Author's Self Archiving Policies, I confirm this Work has not been accepted for publication elsewhere, is not being considered for publication elsewhere and does not duplicate material already published. I confirm all authors consent to publication of this Work and authorise the granting of this licence.

For peer review only

Young adults' perceptions of living with atopic dermatitis in relation to the concept of self-management - a qualitative study.

Short title: Living with eczema in relation to self-management.

Authors: *Susanne Lundin, PhD-student, RN* [a, b], *Marina Jonsson, PhD* [a, c, f], *Carl-Fredrik Wahlgren, MD, PhD, Professor* [d], *Emma Johansson, MD, PhD* [d, e], *Anna Bergström, PhD* [c, f] *& Inger Kull, Professor, PhD* [a, b]

Affiliations:

[a] Department of Clinical Science and Education, Södersjukhuset, Karolinska Institutet, SE-118 67 Stockholm, Sweden.

[b] Sachs' Children and Youth Hospital, Södersjukhuset, SE-118 83 Stockholm, Sweden.

[c] Centre of Occupational and Environmental Medicine, Stockholm County Council, SE-113 65 Stockholm, Sweden.

[d] Dermatology Unit, Department of Medicine, Solna, Karolinska Institutet, SE-171 77 Solna, Sweden.

[e] Dermatology, Theme Inflammation and Infection, Karolinska University Hospital, SE-171 76 Stockholm, Sweden.

[f] Institute of Environmental Medicine, Karolinska Institutet, SE-171 77 Solna, Sweden.

Corresponding Author: Susanne.lundin@ki.se

Susanne Lundin, Department of Clinical Science and Education, Södersjukhuset, Karolinska Institutet, Jägargatan 20, SE-118 67 Stockholm, Sweden, Tel: +46 707 730 124.

Acknowledgement: We thank all the participants in the BAMSE cohort, and all the staff involved in the study through the years.

Patient consent: Not required. All participants were orally and in writing informed that the results of the study would be published in a scientific journal. Details has been removed from case description to ensure anonymity.

Word Count: 3905/4000

For peer review only - http://bmjopen.bmj.com/site/about/guidelines.xhtml

For peer review only

Abstract

Objectives:

Learning to take control of one's health is an important part of the transition from adolescence to adulthood. This study aimed to explore young adults' perceptions of living with atopic dermatitis (AD) in relation to the concept of self-management.

Design:

A qualitative study with an inductive approach was performed through semi-structured interviews (n=15). The interviews were recorded, transcribed verbatim and analysed with systematic text condensation.

Participants:

Young adults (mean age 23,4 y) with persistent atopic dermatitis in a longitudinal population-based birth cohort. To capture experience of living with persistent AD (preschool/school-age onset) of different severity (mild to severe/very severe), a purposive selection was performed. In total 15 young adults were included. Persistent AD (preschool/school-age onset) was defined as dry skin in combination with itchy rash of typical localisation in the 12 months preceding the 16-year and the 24-year follow-ups. Severity was self-assessed using the Patient Oriented Eczema Measure.

Results:

Despite having experience of AD since childhood, the respondents expressed uncertainty about treatment and how it affected their bodies. Their uncertainties and feelings affected how they used topical corticosteroids. The respondents emphasised that they perceived availability of healthcare and knowledge about treatment of AD among healthcare providers to be limited.

For peer review only

For peer review only - http://bmjopen.bmj.com/site/about/guidelines.xhtml

The participants did not state any experiences of support to self-management from healthcare, which affect young adults' possibilities to take full control of their AD care.

Conclusions:

Young adults with preschool/school-age onset of AD are unsure how to treat and manage the disease. One explanation may be insufficient transition process.

Key words: Atopic dermatitis, Interviews, Self-management, Transition, Young adults

Word count, 250/300

For peer review only

For peer review only - http://bmjopen.bmj.com/site/about/guidelines.xhtml

Article summary

Strength and limitation

- Data from a longitudinal population-based birth cohort, with the possibility to obtaining detailed information about different phenotypes and severity of AD over time.
- The AD severity ranged from mild to severe/very severe as, measured by POEM, a validated questionnaire.
- Equal representation of young men and women.
- The results may not be transferred to young adults with more severe atopic dermatitis requiring specialist care.

For peer review only

For peer review only - http://bmjopen.bmj.com/site/about/guidelines.xhtml

Introduction

To optimise self-management in young people with chronic disease, there is a need for a transition process to increase knowledge and to achieve self-care skills and independence [1, 2]. To aid young adults to be autonomous and minimizing dependency from parents and healthcare regarding self-management of eczema, it is important that the transition process starts at an early age (11–13 years) in collaboration with the family and all stakeholders [3]. Atopic dermatitis (AD) [4] is a chronic disease that cannot be cured as yet and requires daily treatment [5]. In addition, it is known that eczema affects health-related quality of life (HRQoL) in both children and adults [6, 7]. The symptoms range from mild to very severe [8]. Most individuals with AD have a mild disease [6, 9].However. there seems to be a discordance between patients' and physicians' assessments of severity, judged using different instruments [10, 11]. The patients reported AD as more severe when their quality of life was impaired, while the physicians reported AD as more severe if sleep was reported to be disturbed [6, 7]. To ensure self-management in AD, it is important that the individuals have sufficient skills related to treatment principles and are aware of the goals of treatment, which are to increase disease control and to extend asymptomatic periods as much as possible [6, 12]. This requires knowledge and education. The mainstay of treatment is use of emollients every day to decrease dryness and extend time to relapse of AD [8]. The first-line treatment of inflammation consists of topical corticosteroids (TCSs) with differing potency depending on age, localisation, and severity of the symptoms [12]. The chronic, recurrent nature of the disease, [6] and the often-time-consuming treatment, to be performed every day, can be a challenge for individuals with AD [13]. Since parents have the main responsibility for the treatment of AD in their children, it is of importance that the parents have been trained to and understand the best management of the disease [8].A shift in responsibility takes place when the children reach adolescence, which requires that the adolescents gradually develop knowledge of their treatment. However,

For peer review only - http://bmjopen.bmj.com/site/about/guidelines.xhtml

results from a Swedish population-based birth cohort shows that many adolescents with AD are untreated [14].

In Sweden, special transition models have been developed for chronic diseases such as congenital heart diseases and diabetes (still under evaluation) [15, 16] but – to the best of our knowledge – not for young persons with AD. According to the recently published guidelines on effective transition by the European Academy of Allergy and Clinical Immunology [3], learning to take control of treatment is an important part of the transition from adolescence to adulthood. Overall experiences of how young adults (from the general population) with AD manage the disease are scarcely studied. The aim of this qualitative study was therefore to explore young adults' perceptions of living with AD in relation to the concept of self-management.

Methods

Design

A qualitative semi-structured interview study with an inductive approach was used to capture young adults' experiences of AD in the concept of self-management. We followed the Consolidated Criteria for Reporting Qualitative Research guideline for conducting and reporting qualitative research [17]

Setting

The present study is a part of the longitudinal population-based cohort BAMSE [18, 19]. A purposive selection was performed to capture young adults with experience of persistent AD (preschool/school-age onset), with various severity (mild to severe/very severe) (Table 1).

For peer review only - http://bmjopen.bmj.com/site/about/guidelines.xhtml

Table 1. Clinical description of sex, severity, and onset of AD among the participants.

Sex	Self-assessed severity at 16 y [1]	Self-assessed severity at 24 y [1]	Onset of eczema up to 4 years [2]	Onset of eczema from 8 years [3]
M	Mild	Moderate		X
F	Mild	Moderate	X	
M	Moderate	Moderate	X	
M	Mild	Moderate	X	
M	Mild	Mild		X
F	Mild	Mild		X
M	Moderate	Severe/very severe	X	
M	Moderate	Mild	X	
F	Mild	Moderate	X	
F	Mild	Severe/very severe	X	
F	Severe/very severe	Moderate	X	
M	Mild	Severe/very severe	X	
F	Moderate	Moderate	X	
F	Mild	Severe/very severe		X
M	Severe/very severe	Severe/very severe	X	

[1] Patient Oriented Eczema Measure class 1, mild eczema (3–7), class 2, moderate eczema (8–16), class 3, severe/very severe eczema (17–28).
[2] Preschool eczema (i.e., eczema onset at 1,2 or 4, not 8, 12 or 16 years of age)
[3] School age eczema (i.e., eczema onset at 8, 12 or 16 not at 1,2 or 4 years of age)

For peer review only

Moreover, we strived to capture an equal proportion of men and women with different severity of AD. They were invited to the interview study by a clinical research nurse over the telephone. Thereafter, the first author contacted the participants, informed them about the study, and asked if they wanted to share their experiences of living with AD.

Inclusion criteria

Young adults with persistent AD, defined as dry skin in combination with itchy rash of typical localisation (neck/throat, arm/leg flexures and/or wrists/ankles) in the 12 months preceding the 16- and the 24-year follow-ups. The severity of AD was self-assessed as mild to severe/very severe using the Patient Oriented Eczema Measure (POEM) [20], which enabled us to capture a range of experiences. The young adults who fulfilled both the questionnaire definition of AD (as above) and the clinical definition of AD; visible AD on the neck/throat and/or flexure surfaces of the arms/legs and/or wrists/ankles and/or reported symptoms of itchy skin in the preceding week as assessed by a trained nurse were included.

Data collection

All interviews were conducted at the research clinic, except two, which were conducted by telephone. The interviews were performed by the first author, an experienced dermatology nurse (SL) with no connection to the study participants before the interviews. The semi-structured interview guide consisted of five themes related to living with AD: daily life, symptoms, treatment, care, and consequences of the disease (Table 2).

For peer review only - http://bmjopen.bmj.com/site/about/guidelines.xhtml

Table 2. Interview guide.

**Themes related to experiences of eczema, treatment and care**	**Follow-up questions**
Tell me what it is like to live with eczema.	Tell me what it has been like lately.<br>What was it like when you were a teenager?<br>Is there any difference?
Tell me what it is like when you have trouble with your eczema.	What is it like when your eczema is not troubling you?<br>How often do you have trouble with your eczema?<br>What do you think is the cause of your eczema?
Tell me how you usually self-treat.	How do you feel it works?<br>How has treatment been shown or explained to you?<br>What do you think about cortisone?<br>What do you think about moisturizing cream?
Tell me about the experiences you have of healthcare in relation to your eczema.	Tell me about the needs you have from healthcare due to your eczema.<br>Tell me about any information you have received from healthcare regarding your eczema.<br>Could it have been given in any other way?
In what situations do you think about your eczema?	Do you avoid doing anything because of your eczema?<br>Have you avoided anything in the past because of your eczema?

For peer review only

Probing questions were used to get a deeper understanding or explanation of some statements. To test the interview guide, one pilot interview was conducted (not included). Minor changes were then made to the interview guide. During the interview, the participants were asked if their answers were interpreted correctly. Thus, the interviewer received confirmation from the respondents. The interviews lasted on average 31 (range 23–43) minutes. Data collection continued until information power was judged to have been achieved. This is an approach which considers the aim, the sample specificity, the theoretical background, the quality of dialogue and the strategy for analyses. Taken together, these determine whether sufficient information power has been obtained. In total, 15 young adults, mean age 23,4 y were included (Table 1), which gave us rich data covering the purpose of the study. All interviews were recorded and transcribed verbatim.

Data analysis

The text was analysed by three researchers (SL, MJ & IK) in collaboration, using systematic text condensation (STC), a thematic cross-case strategy in four steps [21]. First, all interviews were read through (SL, MJ & IK), and preliminary themes were identified based on the contents of the text (SL & MJ). In the second step, meanings units were identified based on the contents of the preliminary themes and code groups were developed (SL & MJ). In the third step, the material from each code group was decontextualized, sorted into subgroups and the contents of the text were condensed (SL, MJ & IK). Lastly, in the fourth step, data were reconceptualised, and categories made based on the essence of the young adults' experiences of eczema (SL, MJ & IK) (Table 3).

For peer review only - http://bmjopen.bmj.com/site/about/guidelines.xhtml

Table 3. The process of analysing young adults' experiences of living with AD with systematic text condensation.

**Step 1.**<br>**Preliminary themes**	**Step2.**<br>**Codes**	**Step 3.**<br>**Subgroups**	**Step 4.**<br>**Categories**
Reminds you	*Always on my mind*<br>*Almost never symptom-free*<br>*As long as it is not visible*	*Strategies*<br>*Acceptance*	*A part of life*
The care is vague	*They do not have time*<br>*Everyone says different things*	*Limited availability*<br>*Limited support*	*Difficult to get help*
	Lack of information<br>*The treatment does not work*	*Uncertainty*<br>*Need for information*	*Limited knowledge*
Physical effects	*Not good for my body*<br>*Being careful*<br>*Burning and stinging*	*Unnatural*<br>*It hurts*	*Impact on my body*

For peer review only

The analysis was inductive, without any theory driven approach to the phenomenon of self-management. We were open to the data and what the material told us, from an individual to a general level. In the analysis process we tried to bracket our preconceptions to minimize the impact of our professional and clinical experiences. Using STC, with many similarities to the editing analysis style [22] we developed, categories derived from the empirical data.

Ethics

The study was approved by the regional ethical review board at Karolinska Institutet, Stockholm (approval number 2016/1380-31-2, 2017/395-32). All participants gave informed consent, 13 with written consent and two by telephone.

Patient and public involvement

The primary intention of this study was to capture young adults' perceptions of living with AD in relation to self-management. However, the young adults were not involved in the design of the study.

Results

The respondents' perceptions of living with AD in the concept of self-management resulted in four categories and eight subgroups (Table 3). Despite having had AD since childhood, the respondents expressed uncertainty about treatment and how it could affect their bodies. Moreover, both symptoms and treatment were described as painful. Their uncertainties and feelings affected how they used TCSs. Though the disease made itself known all the time, most of the participants stated that they had gotten used to living with eczema, it was a part of their life. The respondents emphasised that they experienced that both the accessibility and the support from healthcare providers were limited.

For peer review only

For peer review only - http://bmjopen.bmj.com/site/about/guidelines.xhtml

Limited knowledge

Although most of the respondents had had AD from an early age, they expressed that their knowledge about its cause and treatment was limited. They had created their own treatment routines, based on their experiences and/or what they have learned from their parents. The young adults stated that they experienced a lack of information and directives on treatment.

Uncertainty

The young adults expressed uncertainty about the cause of their AD, how they should treat it, what they should use to treat it, where on the body, and for how long. They had not received much information about the cause or variability of the disease, or about different treatments.

"The doctors have been more like it will go away when you reach a certain age or something, it's just now, it will disappear, so ... I'm just waiting for that age to come" (9)

They felt uncertain about the treatment, if they were doing the right thing, and if the topical treatment should include or consist of cortisone.

"They say cortisone is what helps, I don't know, there's no cortisone in Mometasone, as far as I know" (3)

Need for information.

There was a general belief among respondents that TCSs attenuated or diminished symptoms only for the moment, which reinforced the belief that they could have "become immune" to these creams.

Most of the young adults said they treated themselves regularly, but it was clear they were primarily referring to moisturising creams. The respondents mentioned different treatment recommendations they had been given over the years, such as not to use TCSs around the eyes

For peer review only - http://bmjopen.bmj.com/site/about/guidelines.xhtml

and not to apply cream if it was cold outside. They also reported that they were advised to use TCSs sparingly, at most twice a week, without regard to the severity of the AD.

The respondents expressed that it would be helpful with more information and requested a guide with treatment principles, depending on the severity of the AD. This would be important in order to achieve independence regarding self-management of the disease.

"I have understood by, like, reading and talking with different people that you should have different, like, cycles for it, but I haven't really understood how to do it, because I have not got that input from a doctor, how I should do it" (14)

Impact on my body

Both symptoms and treatment of the AD had a painful impact on the body. Moreover, the need to use different creams to treat the AD was something that felt unnatural and there were thoughts on various treatments adversely affecting the body.

It hurts

Many of the respondents stated that one of the most difficult things about AD was that it hurt. They described the pain as a burning and stinging feeling under the skin. That pain was annoying and could prevent them from doing things that they liked to do. The pain was sometimes more trying than the itching.

"Periodically, it hurt too much to put on socks and shoes…" (1)

They stated that it was painful to apply cream when the AD was severe; they self-treated to get rid of the pain, but that hurt even more.

"It stings, of course, every time I apply the cream" (15)

For peer review only - http://bmjopen.bmj.com/site/about/guidelines.xhtml

Unnatural

Some of the respondents felt that it was unnatural to cover their skin with something that could affect the body negatively, especially in the case of TCSs.

"That you absorb, like, products that the body is not really made to deal with" (2)

They had heard that the skin could become damaged and thinner from using TCSs, which made them anxious; they therefore used these as little as possible. They felt that it was strange to use a treatment that could simultaneously damage the skin even if it decreased the AD.

"I try not to use too much, plain and simple, and hope it is…that I'm like doing what you're supposed to" (7)

Difficult to get help.

There was a perception among the young adults that it was difficult to get help from healthcare, both making an appointment and getting the "right" treatment. The respondents did not state any experiences of education in self-management by healthcare. Moreover, they perceived that healthcare providers did not recognise the symptoms, which resulted in insufficient and delayed treatment.

Limited availability

Even though most of the respondents had had AD all their life, they stated that they had limited contact with healthcare. It seemed to be difficult to get in touch with healthcare and get appropriate help, even when the AD was severe. Sometimes, it took several months to get an appointment and they needed to spend a lot of time trying to get in touch with the care provider. Many of the respondents could not remember the last time they had had an appointment.

For peer review only - http://bmjopen.bmj.com/site/about/guidelines.xhtml

"It was probably in August that I contacted my dermatology department, because it [i.e., eczema] was so very bad…and then I got an appointment for the middle of November" (14)

Limited support

One respondent had experienced that the symptoms of the AD had not been taken seriously; the physician downplayed the respondent's symptoms and compared them with the more widespread symptoms of others. The participants perceived that physicians in general had limited knowledge about treatment of AD and the only advice they had received was to apply topical creams, without further instructions.

"It seems that the GP doesn't know all that much about eczema…and just prescribes ointments that do not help" (8)

Information about how they should treat the eczema also differed from physician to physician.

"Some of them say 'apply it when you need to,' others say, 'apply it like several times a day,' it's like people are guessing a little, they don't have any idea, really" (10)

A part of life

The respondents stated that they had become accustomed to living with AD and had accepted their disease. Even so, they had many strategies about how they should avoid worsening of the AD, which required a lot of routines in the form of regular treatment, avoiding scratching, and creating strategies in daily life.

Strategies

Most of the respondents thought of and were aware of their AD constantly: when it was itching, when it was visible, when they had to apply cream, and so on. They had developed different strategies to avoid worsening of the AD. The respondents perceived it as trying to experience itchiness all the time and they were aware of the importance of not scratching,

For peer review only - http://bmjopen.bmj.com/site/about/guidelines.xhtml

even though it was difficult. Trying not to scratch required constant vigilance. Scratching was the only thing they wanted to do, but they knew it would immediately result in them being "punished", through worsening of the AD.

"That's the main thing in how I handle it, that just sitting there thinking that okay, don't scratch, don't scratch, don't scratch, don't scratch, don't scratch, it has become like a habit to try not to do it" (15)

The major problem was that the AD never disappeared. They had to make choices in their daily life based on their skin condition. Most of the respondents never felt free of symptoms, which they found trying.

"It's probably all the time I'd say, on different parts, it's like it moves around a bit, where it's, like, worst" (10)

The treatment of AD was demanding, according to the respondents. Treatment was a bit awkward and nothing they enjoyed. They had to have a regular routine and a strategy; even then, it took a long time to get rid of the AD and the respondents stated that the creams only helped for the moment.

"But the eczema keeps, like, coming back anyway, even though I have this routine" (9)

They always had to carry creams with them, to school, work, or when they travelled, and if they forgot they were "punished" at once, through worsening of the AD.

"Always having creams with you and applying them is not all that much fun either" (3)

Acceptance

The respondents emphasised that they had gotten used to living with AD and had accepted their situation, even though it was hard. They did not allow AD to keep them from doing things. It was a part of their life.

For peer review only - http://bmjopen.bmj.com/site/about/guidelines.xhtml

"It's, like, something you have, you have to learn to live with it, maybe" (7)

While they had accepted their disease, they tried to avoid revealing their AD, in so far as possible, when it was in visible places on their bodies.

"I do not like it to be visible, so usually when I have these red, I usually use long sleeves, to like cover up" (13)

Discussion

This qualitative interview study of young adults with persistent AD from the general population demonstrated several shortcomings in the concept of self-management. Even though the young adults had experience of persistent AD, they were unsure about how to treat the AD. The respondents expressed limited knowledge about the differences between emollients and TCSs and regarding the potency of the latter. Their uncertainty may be related to their perception of difficulties in getting help from healthcare in the terms of limited knowledge and lack of support about treatment of AD among healthcare providers. While acceptance of the disease was high, the respondents never felt completely symptom-free. Altogether, this indicates an insufficiency regarding self-management, including knowledge and skills in treatment principles for the disease, which may affect young adults' possibilities to take full control of their AD care.

Knowledge about treatment of eczema

The young adults' uncertainty regarding how to treat and manage the AD was in accordance with other studies. The uncertainty seems to be found also in self-treating adults, even if their AD is more severe; this can delay the start of treatment in flare-ups [23]. In contrast, adolescents

For peer review only - http://bmjopen.bmj.com/site/about/guidelines.xhtml

(12-18 y) in a qualitative study [24] expressed satisfaction with the effect of their treatments, even though half of them used TCSs every day because of low efficiency. Moreover, they had limited knowledge and incorrect beliefs about how the treatment worked. The same beliefs were seen in the present study, but the participants in our study expressed less satisfaction and more often believed that treatment, even with emollients, could harm their body.

Factors of importance for self-management

The limited contact with and difficulties in getting help from healthcare can to some extent be due to limited knowledge regarding whom to contact. Healthcare in Sweden is divided into primary care, specialist care, and highly specialised care [25]. Primary care is responsible for treatment and care of the most common skin diseases, such as AD. Individuals with AD can buy up to 30 g of mild TCSs over the counter from a pharmacy; for larger amounts or more potent TGCs, a prescription is needed. The respondents' opinions that physicians had limited knowledge about treatment is in accordance with the results from a qualitative study including GPs. The GPs stated that they used a trial-and-error approach when prescribing emollients, due to the large assortment of different creams, and that they were reluctant to prescribe strong TCSs to children [26]. The prescribed amounts of emollients in primary care have reportedly been unexpectedly low when compared with guidelines, which was also seen for prescription of TCSs in specialist care [27, 28]. To have the possibility to treat AD adequately, the individuals need to have access to appropriate potency and amount of TCSs and to treat the AD daily until the symptoms clear. Thereafter, the TCS therapy should be changed into proactive treatment twice a week [8].

Concerns about side-effects of TCSs are common. Studies including both children and adults with moderate to severe AD have found that approximately 50% of guardians/patients are

For peer review only - http://bmjopen.bmj.com/site/about/guidelines.xhtml

reluctant to use TCSs [29, 30]. In addition, misleading information from relatives/friends, the internet, or pharmacies can lead to poor adherence [31, 32].

Moreover, it has been shown that concerns and uncertainties about side-effects exist even among healthcare providers [33]. Both GPs and pharmacists inform patients about the risks of TCSs and that they should be applied sparingly and not over long periods of time [34]. This can affect the potency and amount of TCSs prescribed and strengthen the feeling among individuals with AD that a TCS is something to be careful with [35, 36].

While the young adults in the present study had gotten used to living with AD, they stated that they needed more support. Furthermore, there seemed to be a need for repeated information about the cause, course, and treatment of the disease. Several studies have stated the importance of shared decision-making, education, and a written action plan [37-40], which could be useful tools to achieve independence. This is important in the process of developing autonomy in self-management and has been confirmed in a study among youths aged 14–24 years [41], which showed that 41% wanted to be more involved in their care. For adolescents with asthma, it is known that support during the transition process results in better adherence to treatment, but the healthcare transition in AD is less studied [42]. Guidelines on asthma emphasise the importance of reviewing each patient's inhalation technique at every healthcare visit, to decrease the risk of exacerbations [43]. It would be equally important to review how patients with AD perform self-treatment, to achieve as long symptom-free periods as possible.

Strengths and limitations

One strength of the present study was that all respondents had well-defined AD based on both prospectively recorded questionnaire data and clinical examinations. Moreover, their AD severity ranged from mild to very severe. Additional strengths are the credibility and

For peer review only

transferability based on the wide range of data from the respondents, which we have exemplified using the respondents' own words.

The respondents were from the general population, which is a strength since most patients with AD are treated in primary care. However, the results may not be transferred to patients with more severe AD, who may have more contact with specialist care. Still, some of the respondents in the present study had moderate to very severe AD.

In qualitative studies, the number of participants is generally low. However, this can be compensated by a purposive selection, to get a deeper understanding of the respondents' experiences. We recruited participants with long experience of mild to very severe AD, who wanted to share their experiences. The data collection gave us varied and rich data on perceptions and experiences of living with AD. An interviewer's preconception about subjects can be both positive and negative. Deep experience can be helpful in the interview situation to promote understanding and when asking probing questions, but can also be negative, leading to assumed understanding of what informants are saying and an assumption that the interviewer and respondent are thinking about the same thing. Preconceptions can also be negative during the analysis phase and the bracketing of the preconceptions is a way to try to avoid these influences. Even so this may be an unattainable goal according to Malterud [44]. To minimise these effects, the analysis was carried out in collaboration with two co-authors with little or no experience in the care of patients with AD.

Conclusions

This qualitative interview study of young adults with AD from the general population demonstrated several shortcomings in self-management. Despite long experience of AD, the young adults expressed limited knowledge and uncertainty about treatment. Their uncertainty may be related to their perception of difficulties in getting help from healthcare and of limited

For peer review only - http://bmjopen.bmj.com/site/about/guidelines.xhtml

support, including knowledge about treatment of AD among healthcare providers. Altogether this may indicate shortcomings in the transition process.

For peer review only

For peer review only - http://bmjopen.bmj.com/site/about/guidelines.xhtml

Authors Contributions: SL, MJ and IK contributed to conception and design of the study. The interviews were managed by SL. The analysis with Systematic Text condensation was conducted by SL, MJ and IK in collaboration. Drafting of the manuscript were conducted by SL with support of IK, C-F. W, EJ and AB. All authors participated in critical revision of the manuscript, provided important intellectual input, and approved the final version.

Conflict of interest: E. Johansson reports personal lecture fees from Sanofi-Genzyme, LEO Pharma, Novartis, and ACO. E. Johansson and S. Lundin has been part of the advisory board at Sanofi-Genzyme. The rest of the authors declare no relevant conflict of interest.

Funding: Funding for this study was provided by the Swedish Asthma and Allergy Association, grant/award number 2017-0007. The funder has not been involved in the design and conduct of the study; collection, management, analysis, and interpretation of the data; preparation, review, or approval of the manuscript; and decision to submit the manuscript for publication.

Data sharing statement: No data are available. Audio files and transcribed text has been deposited anonymously at the BAMSE-secretariat, Institute of Environmental Medicine at Karolinska Institutet, Stockholm, Sweden.

References

1. Gabriel P, McManus M, Rogers K, White P. Outcome Evidence for Structured Pediatric to Adult Health Care Transition Interventions: A Systematic Review. *The Journal of pediatrics*. Sep 2017;188:263-269.e15. doi:10.1016/j.jpeds.2017.05.066
2. Schmidt A, Ilango SM, McManus MA, Rogers KK, White PH. Outcomes of Pediatric to Adult Health Care Transition Interventions: An Updated Systematic Review. *Journal of pediatric nursing*. Mar-Apr 2020;51:92-107. doi:10.1016/j.pedn.2020.01.002
3. Roberts G, Vazquez-Ortiz M, Knibb R, et al. EAACI Guideline on the effective transition of adolescents and young adults with allergy and asthma. *Allergy*. Jun 19 2020;doi:10.1111/all.14459
4. Kantor R, Thyssen JP, Paller AS, Silverberg JI. Atopic dermatitis, atopic eczema, or eczema? A systematic review, meta-analysis, and recommendation for uniform use of 'atopic dermatitis'. *Allergy*. Oct 2016;71(10):1480-5. doi:10.1111/all.12982
5. Bieber T. Atopic dermatitis. *Annals of dermatology*. May 2010;22(2):125-37. doi:10.5021/ad.2010.22.2.125
6. Weidinger S, Novak N. Atopic dermatitis. *Lancet*. Mar 12 2016;387(10023):1109-22. doi:10.1016/S0140-6736(15)00149-X
7. Grant L, Seiding Larsen L, Trennery C, et al. Conceptual Model to Illustrate the Symptom Experience and Humanistic Burden Associated With Atopic Dermatitis in Adults and Adolescents. *Dermatitis : contact, atopic, occupational, drug*. Jul/Aug 2019;30(4):247-254. doi:10.1097/der.0000000000000486
8. Wollenberg A, Barbarot S, Bieber T, et al. Consensus-based European guidelines for treatment of atopic eczema (atopic dermatitis) in adults and children: part I. *Journal of the European Academy of Dermatology and Venereology : JEADV*. May 2018;32(5):657-682. doi:10.1111/jdv.14891

For peer review only - http://bmjopen.bmj.com/site/about/guidelines.xhtml

9. Ballardini N, Kull I, Soderhall C, Lilja G, Wickman M, Wahlgren CF. Eczema severity in preadolescent children and its relation to sex, filaggrin mutations, asthma, rhinitis, aggravating factors and topical treatment: a report from the BAMSE birth cohort. *Br J Dermatol*. Mar 2013;168(3):588-94. doi:10.1111/bjd.12196
10. Wei W, Anderson P, Gadkari A, et al. Discordance Between Physician- and Patient-Reported Disease Severity in Adults with Atopic Dermatitis: A US Cross-Sectional Survey. *American journal of clinical dermatology*. Dec 2017;18(6):825-835. doi:10.1007/s40257-017-0284-y
11. Holm EA, Wulf HC, Stegmann H, Jemec GB. Life quality assessment among patients with atopic eczema. *Br J Dermatol*. Apr 2006;154(4):719-25. doi:10.1111/j.1365-2133.2005.07050.x
12. Weidinger S, Beck LA, Bieber T, Kabashima K, Irvine AD. Atopic dermatitis. *Nature reviews Disease primers*. Jun 21 2018;4(1):1. doi:10.1038/s41572-018-0001-z
13. Santer M, Burgess H, Yardley L, et al. Managing childhood eczema: qualitative study exploring carers' experiences of barriers and facilitators to treatment adherence. *Journal of advanced nursing*. Nov 2013;69(11):2493-501. doi:10.1111/jan.12133
14. Lundin S, Wahlgren CF, Bergstrom A, et al. Use of emollients and topical glucocorticoids among adolescents with eczema - datafrom the population-based birth cohort BAMSE. *Br J Dermatol*. Feb 25 2018;doi:10.1111/bjd.16484
15. Acuña Mora M, Saarijärvi M, Sparud-Lundin C, Moons P, Bratt EL. Empowering Young Persons with Congenital Heart Disease: Using Intervention Mapping to Develop a Transition Program - The STEPSTONES Project. *Journal of pediatric nursing*. Jan-Feb 2020;50:e8-e17. doi:10.1016/j.pedn.2019.09.021
16. Brorsson AL, Bratt EL, Moons P, et al. Randomised controlled trial of a person-centred transition programme for adolescents with type 1 diabetes (STEPSTONES-DIAB): a study protocol. *BMJ open*. Apr 14 2020;10(4):e036496. doi:10.1136/bmjopen-2019-036496
17. Tong A, Sainsbury P, Craig J. Consolidated criteria for reporting qualitative research (COREQ): a 32-item checklist for interviews and focus groups. *International journal for quality in health care : journal of the International Society for Quality in Health Care*. Dec 2007;19(6):349-57. doi:10.1093/intqhc/mzm042
18. Wickman M, Kull I, Pershagen G, Nordvall SL. The BAMSE project: presentation of a prospective longitudinal birth cohort study. *Pediatr Allergy Immunol*. 2002;13 Suppl 15:11-3.
19. Ödling M, Andersson N, Hallberg J, et al. A Gap Between Asthma Guidelines and Management for Adolescents and Young Adults. *The journal of allergy and clinical immunology In practice*. Oct 2020;8(9):3056-3065.e2. doi:10.1016/j.jaip.2020.05.034
20. Charman CR, Venn AJ, Williams HC. The patient-oriented eczema measure: development and initial validation of a new tool for measuring atopic eczema severity from the patients' perspective. *Arch Dermatol*. Dec 2004;140(12):1513-9. doi:10.1001/archderm.140.12.1513
21. Malterud K. Systematic text condensation: a strategy for qualitative analysis. *Scandinavian journal of public health*. Dec 2012;40(8):795-805. doi:10.1177/1403494812465030
22. Crabtree BF, Miller WL. *Doing qualitative research*. SAGE; 1999.
23. Ortiz de Frutos FJ, Torrelo A, de Lucas R, et al. Patient perspectives on triggers, adherence to medical recommendations, and disease control in atopic dermatitis: the DATOP study. *Actas dermo-sifiliograficas*. Jun 2014;105(5):487-96. doi:10.1016/j.ad.2014.01.004
24. Kosse RC, Bouvy ML, Daanen M, de Vries TW, Koster ES. Adolescents' Perspectives on Atopic Dermatitis Treatment-Experiences, Preferences, and Beliefs. *JAMA Dermatol*. Jul 1 2018;154(7):824-827. doi:10.1001/jamadermatol.2018.1096
25. Council SR. The Swedish healthcare system. Updated 2020-02-25. Accessed 2020-07-01,
26. Le Roux E, Powell K, Banks JP, Ridd MJ. GPs' experiences of diagnosing and managing childhood eczema: a qualitative study in primary care. *The British journal of general practice : the journal of the Royal College of General Practitioners*. Feb 2018;68(667):e73-e80. doi:10.3399/bjgp18X694529
27. Choi JY, Dawe R, Ibbotson S, Fleming C, Doney A, Foerster J. Topical treatments in atopic dermatitis: unexpectedly low use of emollients; use of topical corticosteroid is higher in juvenile

For peer review only - http://bmjopen.bmj.com/site/about/guidelines.xhtml

patients, higher in male vs females, and shows independent associations with asthma and depression. *Br J Dermatol*. Jun 30 2019;doi:10.1111/bjd.18265

28. Schmitt J, Schmitt NM, Kirch W, Meurer M. Outpatient care and medical treatment of children and adults with atopic eczema. *Journal der Deutschen Dermatologischen Gesellschaft = Journal of the German Society of Dermatology : JDDG*. Apr 2009;7(4):345-51. doi:10.1111/j.1610-0387.2008.06967.x

29. Zuberbier T, Orlow SJ, Paller AS, et al. Patient perspectives on the management of atopic dermatitis. *J Allergy Clin Immunol*. Jul 2006;118(1):226-32. doi:10.1016/j.jaci.2006.02.031

30. Jung HJ, Bae JY, Kim JE, et al. Survey of disease awareness, treatment behavior and treatment satisfaction in patients with atopic dermatitis in Korea: A multicenter study. *The Journal of dermatology*. Oct 2018;45(10):1172-1180. doi:10.1111/1346-8138.14540

31. Smith SD, Farrugia LL, Harris V, et al. Evaluation of the influence of family and friends, and the Internet on patient perceptions of long-term topical corticosteroid use. *The Journal of dermatological treatment*. Nov 2017;28(7):642-646. doi:10.1080/09546634.2017.1306017

32. Smith SD, Hong E, Fearns S, Blaszczynski A, Fischer G. Corticosteroid phobia and other confounders in the treatment of childhood atopic dermatitis explored using parent focus groups. *The Australasian journal of dermatology*. Aug 2010;51(3):168-74. doi:10.1111/j.1440-0960.2010.00636.x

33. Bos B, Antonescu I, Osinga H, Veenje S, de Jong K, de Vries TW. Corticosteroid phobia (corticophobia) in parents of young children with atopic dermatitis and their health care providers. *Pediatric dermatology*. Jan 2019;36(1):100-104. doi:10.1111/pde.13698

34. Farrugia LL, Lee A, Fischer G, Blaszczynski A, Carter SR, Smith SD. Evaluation of the influence of pharmacists and GPs on patient perceptions of long-term topical corticosteroid use. *The Journal of dermatological treatment*. Mar 2017;28(2):112-118. doi:10.1080/09546634.2016.1213353

35. Arkwright PD, Motala C, Subramanian H, Spergel J, Schneider LC, Wollenberg A. Management of difficult-to-treat atopic dermatitis. *The journal of allergy and clinical immunology In practice*. Mar 2013;1(2):142-51. doi:10.1016/j.jaip.2012.09.002

36. Feldman SR, Vrijens B, Gieler U, Piaserico S, Puig L, van de Kerkhof P. Treatment Adherence Intervention Studies in Dermatology and Guidance on How to Support Adherence. *American journal of clinical dermatology*. Apr 2017;18(2):253-271. doi:10.1007/s40257-017-0253-5

37. Noerreslet M, Jemec GB, Traulsen JM. Involuntary autonomy: patients' perceptions of physicians, conventional medicines and risks in the management of atopic dermatitis. *Social science & medicine (1982)*. Nov 2009;69(9):1409-15. doi:10.1016/j.socscimed.2009.08.036

38. Stalder JF, Bernier C, Ball A, et al. Therapeutic patient education in atopic dermatitis: worldwide experiences. *Pediatric dermatology*. May-Jun 2013;30(3):329-34. doi:10.1111/pde.12024

39. Muller SM, Tomaschett D, Euler S, Vogt DR, Herzog L, Itin P. Topical Corticosteroid Concerns in Dermatological Outpatients: A Cross-Sectional and Interventional Study. *Dermatology (Basel, Switzerland)*. 2016;232(4):444-52. doi:10.1159/000446068

40. Shi VY, Nanda S, Lee K, Armstrong AW, Lio PA. Improving patient education with an eczema action plan: a randomized controlled trial. *JAMA Dermatol*. Apr 2013;149(4):481-3. doi:10.1001/jamadermatol.2013.2143

41. Schuiteman S, Chua KP, Plegue MA, Ilyas O, Chang T. Self-Management of Health Care Among Youth: Implications for Policies on Transitions of Care. *The Journal of adolescent health : official publication of the Society for Adolescent Medicine*. Feb 26 2020;doi:10.1016/j.jadohealth.2020.01.009

42. Vazquez-Ortiz M, Angier E, Blumchen K, et al. Understanding the challenges faced by adolescents and young adults with allergic conditions: a systematic review. *Allergy*. Mar 6 2020;doi:10.1111/all.14258

43. GINA gifa. Pocket guide for asthma management and prevention (for adults and children older than 5 years). Updated 2020-02-28.

44. Malterud K. *Kvalitativa metoder i medicinsk forskning : en introduktion (Qualitative methods in medical research: an introduction)*. Studentlitteratur; 2014.

For peer review only - http://bmjopen.bmj.com/site/about/guidelines.xhtml

Manuscript: Clinicians' perspectives of parental decision-making following diagnosis of a severe congenital anomaly: qualitative study.

Consolidated criteria for reporting qualitative studies (COREQ): 32-item checklist

Developed from:
Tong A, Sainsbury P, Craig J. Consolidated criteria for reporting qualitative research (COREQ): a 32-item checklist for interviews and focus groups. *International Journal for Quality in Health Care*. 2007. Volume 19, Number 6: pp. 349 – 357

No. Item	Guide questions/description	Reported on Page #
**Domain 1: Research team and reflexivity**		
Personal Characteristics		
1. Inter viewer/facilitator	Which author/s conducted the inter view or focus group?	Page 8
2. Credentials	What were the researcher's credentials? E.g. PhD, MD	Page 8
3. Occupation	What was their occupation at the time of the study?	Page 8
4. Gender	Was the researcher male or female?	Page 1and 8
5. Experience and training	What experience or training did the researcher have?	Page 8
Relationship with participants		
6. Relationship established	Was a relationship established prior to study commencement?	**No** .
7. Participant knowledge of the interviewer	What did the participants know about the researcher? e.g. personal goals, reasons for doing the research	At start of the interviews the author introduce herself as RN and PhD-student
8. Interviewer characteristics	What characteristics were reported about the inter viewer/facilitator? e.g. Bias, assumptions, reasons and interests in the research topic	Page 8 and 21

**Domain 2: study design**		
Theoretical framework		
9. Methodological orientation and Theory	What methodological orientation was stated to underpin the study? e.g. grounded theory, discourse analysis, ethnography, phenomenology, content analysis	Page 6
Participant selection		
10. Sampling	How were participants selected? e.g. purposive, convenience, consecutive, snowball	Page 6
11. Method of approach	How were participants approached? e.g. face-to-face, telephone, mail, email	Page 8
12. Sample size	How many participants were in the study?	Page 10
13. Non-participation	How many people refused to participate or dropped out? Reasons?	Page 6
Setting		
14. Setting of data collection	Where was the data collected? e.g. home, clinic, workplace	Page 8 .
15. Presence of non-participants	Was anyone else present besides the participants and researchers?	No
16. Description of sample	What are the important characteristics of the sample? e.g. demographic data, date	Page 8
Data collection		
17. Interview guide	Were questions, prompts, guides provided by the authors? Was it pilot tested?	Table 2. Page 8 and 10
18. Repeat interviews	Were repeat inter views carried out? If yes, how many?	No
19. Audio/visual recording	Did the research use audio or visual recording to collect the data?	Page 10
20. Field notes	Were field notes made during and/or after the inter view or focus group?	No
21. Duration	What was the duration of the inter views	Page 10

For peer review only

	or focus group?	
22. Data saturation	Was data saturation discussed?	Page 10
23. Transcripts returned	Were transcripts returned to participants for comment and/or correction?	No
**Domain 3: analysis and findings**		
Data analysis		
24. Number of data coders	How many data coders coded the data?	Page 12
25. Description of the coding tree	Did authors provide a description of the coding tree?	Page 10 and Table 3
26. Derivation of themes	Were themes identified in advance or derived from the data?	Page 12
27. Software	What software, if applicable, was used to manage the data?	Not applicable
28. Participant checking	Did participants provide feedback on the findings?	No
Reporting		
29. Quotations presented	Were participant quotations presented to illustrate the themes/findings? Was each quotation identified? e.g. participant number	Page 12-18
30. Data and findings consistent	Was there consistency between the data presented and the findings?	Page 12-18
31. Clarity of major themes	Were major themes clearly presented in the findings?	Page 12-18
32. Clarity of minor themes	Is there a description of diverse cases or discussion of minor themes?	-

For peer review only
